# Assessment of suicide attempt and death in bipolar affective disorder: a combined clinical and genetic approach

**DOI:** 10.1038/s41398-021-01500-w

**Published:** 2021-07-07

**Authors:** Eric T. Monson, Andrey A. Shabalin, Anna R. Docherty, Emily DiBlasi, Amanda V. Bakian, Qingqin S. Li, Douglas Gray, Brooks Keeshin, Sheila E. Crowell, Niamh Mullins, Virginia L. Willour, Hilary Coon

**Affiliations:** 1 Department of Psychiatry & Huntsman Mental Health Institute, University of Utah, Salt Lake City, UT USA; 2 Department of Family and Preventative Medicine, Division of Public Health, University of Utah, Salt Lake City, UT USA; 3 Neuroscience Therapeutic Area, Janssen Research and Development, Titusville, NJ USA; 4 VISN 19 Rocky Mountain MIRECC, George E. Wahlen VA Medical Center, Salt Lake City, UT USA; 5 Department of Pediatrics, University of Utah, Salt Lake City, UT USA; 6 Department of Psychology, University of Utah, Salt Lake City, UT USA; 7 Department of Obstetrics and Gynecology, University of Utah, Salt Lake City, UT USA; 8 Department of Psychiatry, Icahn School of Medicine at Mount Sinai, New York City, NY USA; 9 Department of Genetics and Genomic Sciences, Icahn School of Medicine at Mount Sinai, New York City, NY USA; 10 Department of Psychiatry, University of Iowa, Iowa City, IA USA; 11 Department of Biomedical Informatics, University of Utah, Salt Lake City, UT USA; 12 Division of Genetic Epidemiology, University of Utah, Salt Lake City, UT USA

**Keywords:** Comparative genomics, Bipolar disorder

## Abstract

Bipolar disorder (BP) suicide death rates are 10–30 times greater than the general population, likely arising from environmental and genetic risk factors. Though suicidal behavior in BP has been investigated, studies have not addressed combined clinical and genetic factors specific to suicide death. To address this gap, a large, harmonized BP cohort was assessed to identify clinical risk factors for suicide death and attempt which then directed testing of underlying polygenic risks. 5901 individuals of European ancestry were assessed: 353 individuals with BP and 2498 without BP who died from suicide (BPS and NBPS, respectively) from a population-derived sample along with a volunteer-derived sample of 799 individuals with BP and a history of suicide attempt (BPSA), 824 individuals with BP and no prior attempts (BPNSA), and 1427 individuals without several common psychiatric illnesses per self-report (C). Clinical and subsequent directed genetic analyses utilized multivariable logistic models accounting for critical covariates and multiple testing. There was overrepresentation of diagnosis of PTSD (OR = 4.9, 95%CI: 3.1–7.6) in BPS versus BPSA, driven by female subjects. PRS assessments showed elevations in BPS including PTSD (OR = 1.3, 95%CI:1.1–1.5, versus C), female-derived ADHD (OR = 1.2, 95%CI:1.1–1.4, versus C), and male insomnia (OR = 1.4, 95%CI: 1.1–1.7, versus BPSA). The results provide support from genetic and clinical standpoints for dysregulated traumatic response particularly increasing risk of suicide death among individuals with BP of Northern European ancestry. Such findings may direct more aggressive treatment and prevention of trauma sequelae within at-risk bipolar individuals.

## Introduction

Suicidal behavior, which can be defined in many ways, is here defined as behaviors that include suicide attempt and death by suicide [1]. Prior suicide attempt is consistently one of the strongest predictors of eventual death by suicide [2, 3]. However, the vast majority of individuals that attempt suicide will not die by suicide. Only ~2.8% of individuals with at least one prior suicide attempt die by suicide [4]. Despite this, existing research on suicidal behavior primarily focuses on the evaluation of suicide attempt under the assumption that attempt acts as an adequate proxy for suicide death. Distinguishing factors important to suicide attempt versus suicide death will be crucial to the implementation of effective interventions to those most likely to die.

Patients with bipolar disorder (BP) have high rates of suicide attempt (30–50%) and death (15–20%) [5–7]. The rates for attempt and death are approximately twice those seen for major depression [5, 8] and the rate of death is greater than in any disorder except schizophrenia [9]. These features suggest potential elevation of biological risk of suicide specific to BP. For this study, we leveraged the largest cohort of population-ascertained suicide decedents available, representing over 7000 individuals collected over two decades in the state of Utah [10]. The majority of these subjects have genetic data available via array genotyping. Electronic health records (EHRs) of these subjects allowed for the identification of 353 individuals with BP who died by suicide. In addition to this unique sample, we utilized a large array-genotyped NIMH Genetics Initiative sample (*N* = 3050) including individuals with diagnosed BP, with and without a history of suicide attempt, and a comparison group that was screened for several common psychiatric illnesses via self-report [11–14]. Together, these cohorts allowed a comprehensive study of clinical and hypothesis-driven genetic risk factors for suicide death in bipolar disorder and allowed for differentiation of risk factors between attempt and death.

## Materials and methods

### Sample selection

Two distinct sample sets were utilized. The first was composed of >7000 population-ascertained individuals who died from suicide from the Utah Suicide Genetics Research Study (USGRS). These samples were collected through a collaboration with the Utah Medical Examiner’s office and have been securely linked to electronic health record (EHR) information via the Utah Population Database (UPDB), a statewide data resource of demographic and health information (https://healthcare.utah.edu/huntsmancancerinstitute/research/updb). Because the study design involved analysis of EHR followed by hypothesis-driven analyses of polygenic risk, a subset of 2851 suicide deaths with screened genome-wide genotyping data and who were linked to existing EHR data (see supplementary methods) were retained. Inpatient and ambulatory EHRs were obtained from Utah providers covering approximately 85% of the state, though they may not represent all health records for each individual. After EHR linking, identifying data were stripped before providing data to the research team; suicide cases were referenced by anonymous IDs. The individuals selected for this study had at least one prior diagnostic code for bipolar disorder (specifically bipolar I or bipolar NOS). EHR were also screened for schizophrenia diagnoses and these Individuals were removed from the BP group to increase the probability of diagnostic homogeneity. All individuals excluded from diagnosis in the BP suicide death group were also excluded from the non-BP suicide deaths to ensure that no known BP diagnoses (including diagnoses of cyclothymia, manic depressive disorder, and bipolar II) would be present within the non-BP suicide death group. A total of 353 individuals with BP who died from suicide (referred to as “BPS”) and 2498 individuals without a diagnosis of BP who died from suicide (referred to as “NBPS”) from Utah were included in the analyses. This sample represents the single largest known genotyped sample of BP suicide deaths and, in a post-hoc evaluation of power, was predicted to have 80% power to identify a clinical diagnostic difference between suicide death and comparison groups with an odds ratio of 1.7 as calculated via the UCSF online sample size calculator [15].

The second set of individuals was composed of a pre-existing de-identified genotyped dataset derived from the NIMH genetics initiative bipolar GWAS [11–13, 16] with a history of bipolar I or schizoaffective, bipolar type diagnoses as determined by formal evaluation and best-estimate diagnosis meeting criteria from the DSM-IIIR [17] or DSM-IV [18]. These individuals were selected for having complete clinical information from interview evaluation (individuals with missing information were excluded from this study). It is noted that interviews did not systematically evaluate for all included diagnoses, and that information from collected medical records and family informants that were used to support diagnoses were not available for all subjects (see Supplementary materials for more specific details of collected subject data). Individuals with a diagnosis of bipolar disorder and a history one or more suicide attempts (*N* = 799, referred to as “BPSA”) and individuals with a diagnosis of BP and no history of prior suicide attempt (*N* = 824, referred to as “BPNSA”) were selected for inclusion in the study with diagnostic and historical data being obtained via the Diagnostic Interview for Genetic Studies (DIGS v4) [19]. A comparison group of individuals who were screened for several common psychiatric disorders via self-report [14] were also included (*N* = 1427, referred to as “C”). Briefly, screened illnesses excluded at the time of sample construction included major depression, psychosis, and bipolar disorder [12]. This sample has been described elsewhere (including acquisition and quality control efforts), noting that informed consent and appropriate IRB approval for all involved subjects was obtained in the original studies [11–14].

### Genotyping

Utah suicide decedent DNAs were extracted from whole blood, and were genotyped using the Illumina Infinium PsychArray (https://www.illumina.com/products/by-type/microarray-kits/infinium-psycharray.html) as described elsewhere [10]. DNAs from the NIMH BP and control populations were extracted from lymphoblastoid cell lines maintained at the NIMH DNA Repository (Infinite biologics, Rutgers RUCDR, https://www.rucdr.org/), and were genotyped using the Affymetrix Genome-Wide Human SNP Array 6.0 (https://www.thermofisher.com/us/en/home/life-science/microarray-analysis/affymetrix.html) and processed as previously described [12, 16, 20,]. Shared high-quality called variants from both platforms were combined and imputed via the Michigan Imputation Server [21] to a total of 7 437 997 high quality imputed variants. Extensive quality control steps, including assessment for ancestry and relatedness, were utilized to prepare this sample for analysis (see Supplementary methods). Due to sensitivity of polygenic risk scores to ancestry effects, this study focused only on individuals of >90% European ancestry.

### Analysis of sample characteristics

Statistical evaluation of the distribution of sex, age, education level, and clinical categories across all comparison groups were evaluated by chi-square (sex, education, clinical categories) or ANOVA (age).

### Analyses of clinical data

Five clinical diagnostic categories were constructed from available diagnoses based on consensus of M.D./Ph.D.-level clinicians (E.M., B.K., A.D.) for all subjects with full details within the Supplementary methods and Supplementary Table S1. Briefly, these categories represented non-traumatic anxiety disorders, behavioral disorders, personality disorders, eating disorders, and PTSD. The primary clinical analysis compared BPS, BPSA, and BPNSA within these categories.

All clinical categories were also secondarily evaluated to determine if observed effects were specific to BPS. NBPS were compared with BPS for these assessments.

All clinical analyses utilized logistic multivariable regression in R [22] accounting for age, sex, and education level. All variables were derived from single, independent measures for each subject. It was also noted that BPS had considerably more clinical diagnoses, on average, than NBPS, necessitating the inclusion of a clinical diagnosis count covariate within analyses comparing these groups. Effect size estimates were calculated via adjusted odds ratio from each model. Correction for multiple testing of 15 primary and 30 sex-specific clinical analyses utilized the Benjamini–Hochberg method with a false discovery rate of 0.05.

### Analyses of genetic data

PRS calculations of several phenotypes, selected via significant clinical analysis results, were generated from 9 GWAS datasets (19 PRS total). Summary GWAS data arose from meta-analyses with publicly available summary statistics for ADHD [23, 24], anxiety [25], insomnia [26], PTSD [27], suicide attempt [28], and neuroticism [29]. Many of these sets included summary statistics for population subsets, including male- and female-only analyses, referred to here as “sex-derived” sets, which were also analyzed. Suicide death PRS was calculated from the USGRS suicide death GWAS using a cross-validation approach described elsewhere [10]. Suicide attempt in bipolar disorder PRS were calculated from published Psychiatric Genomic Consortium data [28] with all overlapping study subjects removed. PRS calculations were conducted using PRSice 2.0 [30] with a *p*-value threshold of 1.0 as described in the Supplemental note. All PRS were standardized to Z-scores prior to statistical analysis.

Pairwise comparisons of BPS, BPSA, BPNSA, and C utilized multivariable logistic regression models in R [22], accounting for age, sex, and the first 10 principal components to control for residual ancestry effects. As with the clinical variables and covariates, all variables were obtained from independent measures without duplication. PRS measures were evaluated to have similar variance across groups during assessment and as visualized in plots. Effect size estimates were calculated by adjusted odds ratio from each model. Correction for multiple testing of 114 primary and 204 sex-specific PRS analyses utilized Benjamini–Hochberg calculations with a false discovery rate of 0.05.

## Results

### Sample evaluation

Sample demographics, including frequency of comorbid diagnoses within the defined clinical categories and statistical evaluation of the distribution across the groups for each demographic are outlined in Table 1. It is noted that the groups varied from one another significantly, but particularly striking differences can be appreciated in the sex distribution of each group. These differences are consistent with expectations that more males than females die from suicide, and more females than males attempt suicide [31]. However, it is notable that the excess of male deaths was significantly lower in BPS when compared to NBPS (62.0% of BPS being male versus 79.5% of NBPS being male, OR = 0.42, 95%CI = 0.33–0.53; *X*^2^ = 54.1, *P* = 1.9 × 10^–13^).

**Table 1:** Study sample demographics by comparison group.

Base demographics	BPS	NBPS	BPSA	BPNSA	C	*P*
Total Subjects	353	2 498	799	824	1 427	
Males	219 (62.0%)	1 987 (79.5%)	262 (32.8%)	391 (47.5%)	765 (53.6%)	<0.0001
Females	134 (38.0%)	511 (20.5%)	537 (67.2%)	433 (52.5%)	662 (46.4%)	
Mean Age	39.5	43.2	41.8	41.6	52.3	0.0001
*Education level equivalent*						
8th Grade or lower	3 (0.8%)	55 (2.2%)	14 (1.7%)	10 (1.2%)	N/A	<0.0001
9th to 12th Grade, No Grad	39 (11.0%)	305 (12.2%)	54 (6.8%)	37 (4.5%)	N/A	
HS Grad/GED	117 (33.1%)	912 (36.5%)	131 (16.4%)	142 (17.2%)	N/A	
Some college, no degree	100 (28.3%)	602 (24.1%)	89 (11.1%)	74 (8.9%)	N/A	
Associates	29 (8.2%)	203 (8.1%)	142 (17.1%)	118 (14.3%)	N/A	
Bachelors	40 (11.3%)	240 (9.6%)	247 (30.9%)	270 (32.8%)	N/A	
Masters	13 (3.7%)	107 (4.2%)	97 (12.1%)	144 (17.5%)	N/A	
PhD or higher	7 (2.0%)	40 (1.6%)	13 (1.6%)	18 (2.2%)	N/A	
*Clinical categories*						
Non-traumatic anxiety disorders	236 (66.9%)	965 (29.5%)	446 (55.8%)	388 (47.1%)	N/A	<0.0001
Behavioral disorders	93 (26.3%)	207 (6.3%)	196 (24.5%)	146 (17.7%)	N/A	<0.0001
Eating disorders	13 (3.7%)	15 (0.4%)	144 (18.0%)	89 (10.8%)	N/A	<0.0001
PersoNALITY DIsorders	96 (27.2%)	160 (4.9%)	66 (8.3%)	46 (5.6%)	N/A	<0.0001
Post-traumatic stress disorders	77 (21.8%)	154 (4.7%)	50 (6.3%)	59 (7.2%)	N/A	<0.0001
Group Key						
BPS definition:	individuals with bipolar disorder who died by suicide					
NBPS definition:	individuals without a diagnosis of bipolar disorder who died from suicide					
BPSA definition:	individuals with bipolar disorder who have a history of one or more suicide attempts					
BPNSA definition:	individuals with bipolar disorder who have no history of a suicide attempt					
C definition:	Comparison group of Individuals without several common psychiatric diagnoses based on self-report [14]					

### Clinical analyses of BPS, BPSA, and BPNSA

**Fig. 1:**
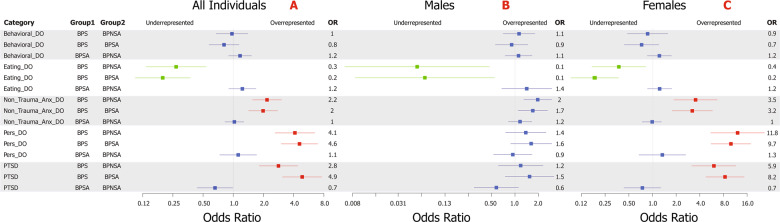
BPS versus BPSA and BPNSA clinical category analysis results. Forest plot distribution of corrected odds ratios of the primary clinical category comparisons (with 95% confidence interval represented by whiskers) within all individuals (**A**), males (**B**), and females (**C**). Labeling of comparison groups is as follows: BPS = individuals with bipolar disorder who died by suicide, BPSA = individuals with bipolar disorder who have a history of one or more suicide attempts, and BPNSA = individuals with bipolar disorder who have no history of a suicide attempt. Significant results are colored with overrepresentation shown in red and underrepresentation in green. Non-significant results are shown in blue. Results were corrected for multiple testing via the Benjamini–Hochberg method with an FDR of 0.05 for a total of 15 tests in the primary analysis (**A**) and 30 in the sex-specific analyses (**B**, **C**) and for critical covariates.

Complete results can be viewed within Supplementary Tables S2 and S3 with odds ratios and confidence intervals displayed in Fig. 1. All results are corrected for multiple testing and covariates. BPS versus BPSA showed overrepresentation for diagnoses of PTSD (OR = 4.9, 95%CI = 3.1–7.6; *P* = 6.0 × 10^−11^), personality disorders (OR = 4.6, 95%CI = 3.0–7.0; *P* = 2.2 × 10^−11^; noting the caveat discussed in the limitations), and non-traumatic anxiety disorders (OR = 2.0, 95%CI = 1.4–2.8; *P* = 1.3 × 10^−4^). Eating disorder diagnoses were significantly reduced within BPS versus BPSA (OR = 0.2, 95%CI = 0.1–0.4; *P* = 2.2 × 10^−6^). No comparisons were significant between BPSA and BPNSA.

Secondary sex-specific results are shown in Fig. 1B and C. Females strongly drove overrepresentations of PTSD (OR = 8.2, 95%CI = 4.7–14.4; *P* = 3.4 × 10^−11^), personality disorder (OR = 9.7, 95%CI = 5.5–17.4; *P* = 4.5 × 10^−13^), and non-traumatic anxiety disorder (OR = 3.2, 95%CI = 1.7–5.7; *P* = 6.1 × 10^−4^) in BPS versus BPSA. Females also drove the reduced rate of eating disorder diagnoses in BPS versus BPSA (OR = 0.2, 95%CI = 0.1–0.4; *P* = 1.1 × 10^−5^). Though male BPS versus male BPSA showed nominal overrepresentation of non-traumatic anxiety disorders, personality disorders, and PTSD, none of these results survived correction for multiple testing.

### Clinical analysis of BPS versus NBPS

**Fig. 2:**
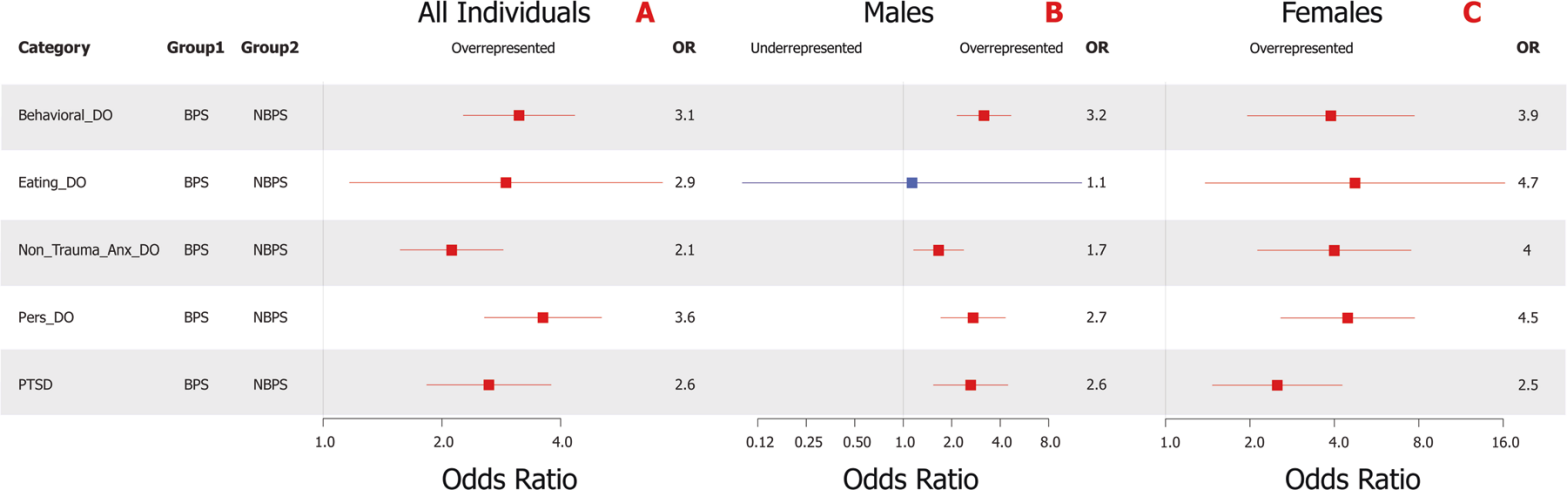
BPS versus NBPS clinical category analysis results. Forest plot distribution of corrected odds ratios of the suicide-only clinical category comparisons (with 95% confidence interval represented by whiskers) within all subjects (**A**), male subjects (**B**), and female subjects (**C**). Labeling of comparison groups is as follows: BPS = individuals with bipolar disorder who died by suicide and NBPS = individuals without a diagnosis of bipolar disorder who died from suicide. Significant results are colored with overrepresentation shown in red and underrepresentation in green. Non-significant results are shown in blue. Results were corrected for multiple testing via the Benjamini–Hochberg method with an FDR of 0.05 for a total of 5 tests in the primary analysis (**A**) and 10 in the sex-specific analyses (**B**, **C**) and for critical covariates.

Comparisons are shown in Fig. 2 with complete results in Supplementary Tables S4 and S5. Even after correction for medical record completeness and years of education, BPS were elevated versus NBPS for all comorbid psychiatric diagnoses, including within sex-specific analyses (except for the male eating disorders comparison). The strongest elevations were noted within personality disorders (OR = 3.6, 95%CI = 2.6–5.1; *P* = 9.1 × 10^−13^) and behavioral disorders (OR = 3.1, 95%CI = 2.3–4.3; *P* = 1.3 × 10^−11^). In addition, findings show similar effect sizes within the sex-specific comparisons (Fig. 2B and C), regardless of sex, though all diagnoses were seen at higher frequencies within females, both within the BPS and NBPS.

### Genetic risk analysis across BPS, BPSA, BPNSA, and C

**Fig. 3:**
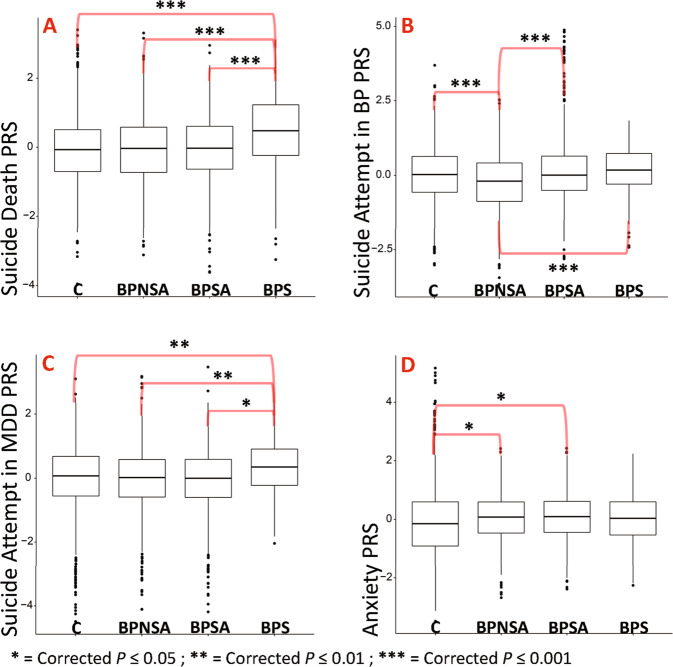
Suicide death, attempt, and anxiety PRS results. Box plot representations of the top findings from polygenic risk score association testing. Each plot represents comparison group (*x*-axis) versus standardized polygenic risk score for the given phenotype (*y*-axis). **A** Suicide death PRS. **B** Suicide attempt in bipolar disorder PRS. **C** Suicide attempt in MDD PRS. **D** Anxiety PRS. Comparison group definitions: BPS = individuals with bipolar disorder who died by suicide, BPSA = individuals with bipolar disorder who have a history of one or more suicide attempts, BPNSA = individuals with bipolar disorder who have no history of a suicide attempt, and C = comparison individuals without several common psychiatric illnesses per self-report [14]. Selected results shown; all displayed results have been corrected for multiple testing (Benjamini–Hochberg method with FDR of 0.05 correcting for 114 tests for all displayed results) and account for critical covariates. All shown results arose from evaluating all subjects (male and female).

**Fig. 4:**
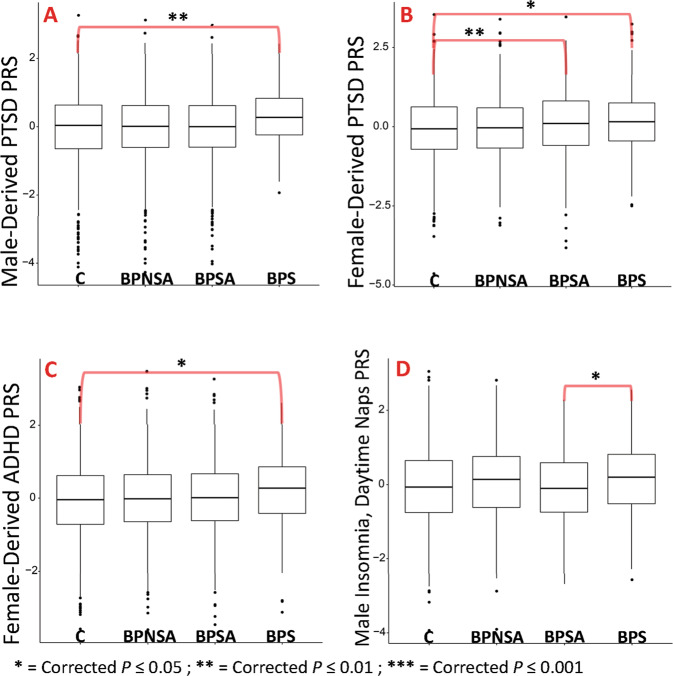
PTSD and behavioral PRS results. Box plot representations of the top findings from polygenic risk score association testing. Each plot represents comparison group (*x*-axis) versus standardized polygenic risk score for the given phenotype (*y*-axis). **A** Male-derived PTSD PRS. **B** Female-derived PTSD PRS. **C** Female-derived ADHD PRS. **D** Male only (sex-specific) insomnia (daytime napping subgroup) PRS. Comparison group definitions: BPS = individuals with bipolar disorder who died by suicide, BPSA = individuals with bipolar disorder who have a history of one or more suicide attempts, BPNSA = individuals with bipolar disorder who have no history of a suicide attempt, and C = comparison individuals without several common psychiatric illnesses per self-report [14]. Selected results shown; all displayed results have been corrected for multiple testing (Benjamini–Hochberg method with FDR of 0.05 correcting for 114 tests for **A**–**C** and 204 tests for **D**) and account for critical covariates. Note that sex-derived refers to PRS calculated based on weighted results from the given sex in the original GWAS. All results were evaluated from all (male and female) subjects in the current study with the exception of **D**, which was an evaluation of only males.

See Figs. 3 and 4 with full results in Supplementary Tables S6 and S7.

i. Suicide attempt in MDD and BP and suicide death PRS.
  Suicide death PRS (Fig. 3A) was elevated in BPS versus BPSA (OR = 1.6, 95%CI = 1.4–1.9; *P* = 7.8 × 10^−10^). Suicide attempt in BP PRS (Fig. 3B) showed elevations in BPS versus BPNSA (OR = 1.5, 95%CI = 1.3–1.8; *P* = 1.9 × 10^−6^) and BPSA versus BPNSA (OR = 1.5, 95%CI = 1.3–1.6; *P* = 1.1 × 10^−11^). Suicide attempt in BP was also reduced in BPNSA versus C (OR = 0.7, 95%CI = 0.7–0.8; *P* = 4.2 × 10^−8^). PRS for suicide attempt within major depressive disorder (Fig. 3C) was elevated in BPS compared with all comparison groups, including versus BPSA (OR = 1.3, 95%CI = 1.1–1.6; *P* = 1.8 × 10^−2^).
ii. Anxiety, and neuroticism PRS.
  Anxiety (Fig. 3D; OR = 1.2, 95%CI = 1.1–1.3, *P* = 1.8 × 10^−2^) and neuroticism (worry subcluster, not shown; OR = 1.2, 95%CI = 1.1–1.3, *P* = 6.0 × 10^−3^) showed elevated PRS in BPSA versus C. Notably, PRS for anxiety and neuroticism also showed either significant or nominal elevation in BPS, BPSA, and BPNSA versus C, and ad-hoc comparisons of BPS, BPSA, and BPNSA combined into a single comparison group versus C showed *P* = 5.6 × 10^–5^ for anxiety and 3.8 × 10^−4^ for neuroticism.
iii. PTSD and behavioral PRS.
  The PTSD GWAS published summary statistics for males, females, and all subjects [27]. All-subject (not shown; OR = 1.3, 95%CI = 1.1–1.5, *P* = 7.8 × 10^−3^) and male-derived PTSD PRS (Fig. 4A; OR = 1.3, 95%CI = 1.1–1.5, *P* = 8.0 × 10^−3^) were elevated within BPS versus C. Female-derived PTSD PRS (Fig. 4B), however, was elevated in both BPSA versus C (OR = 1.2, 95%CI = 1.1–1.3, *P* = 3.6 × 10^−3^) and BPS versus C (OR = 1.2, 95%CI = 1.1–1.4, *P* = 2.5 × 10^−2^). None of these comparisons remained significant in sex-specific analyses, but effect sizes were similar.
  Female-derived ADHD PRS (Fig. 4C) was elevated within BPS versus C (OR = 1.2, 95%CI = 1.1–1.4, *P* = 2.0 × 10^−2^).
iv. Sex-specific PRS.
  Sex-specific PRS analyses (Supplementary Table S7) generally reproduced findings with similar effect sizes in both sexes, but often did not survive correction for multiple testing in the setting of smaller comparison groups. One new finding was identified, however: male-specific polygenic risk for insomnia (daytime napping subgroup, Fig. 4D) was elevated in BPS versus BPSA with OR = 1.4, 95%CI = 1.1–1.7, *P* = 4.3 × 10^−2^.
v. BPS versus NBPS PRS.
  PRS analyses of BPS versus NBPS (Supplementary Tables S8–S9) showed no significant differences between groups, including within sex-specific analyses.

## Discussion

Suicide attempt is often used as a proxy for suicide death and, as such, frequently serves as the primary phenotype within studies of suicide. Suicide attempts and deaths, however, are separate groups that overlap. The unique resource of the USGRS dataset allowed more thorough exploration of suicide death within BP, identifying several potentially important clinical and genetic associations that may aid in identifying and differentiating those at highest risk for suicide attempt and death.

### The role of trauma and its enduring effects in suicidal behavior

This study is the first, to our knowledge, to identify combined clinical and genetic evidence of factors that may distinguish risk for suicide death from attempt in BP. Specifically, PTSD and personality disorder diagnoses were strongly elevated in BPS versus BPSA. In addition, clinically informed genetic analyses identified elevated polygenic risk for PTSD in BPS. Trauma, and subsequent response, is a common factor in these findings. A history of trauma is required for PTSD [32] and is correlated with a more severe course [33] with an increased risk for suicidal behavior in BP [34, 35]. Trauma is also frequently present in personality disorders [32] which are associated with suicidal behavior [36] and often comorbid with PTSD [37].

Such findings may also provide unifying support for the role of stress response pathways such as the hypothalamic pituitary axis (HPA) [38]. Prior evidence suggests that genetic disruption of the HPA-axis may interact with trauma/severe stress exposure to increase risk for suicide attempt [39]. This study provides novel evidence that traumatic disruption may increase risk of death from suicide. Indeed, recent evidence has arisen that early-life traumatic exposure in the setting of elevated polygenic risk for BP is significantly correlated with an increase in suicide attempts [40]. Taken together, the clinical and genetic findings of this study support the long-standing stress-diathesis model for suicidal behavior in BP [41], and specifically extend those findings to risk for suicide death.

Data from this study also demonstrate potentially important sex-related differences, with trauma-related diagnoses being driven by female BPS. In contrast, males and female BPS demonstrate relatively equal effects within polygenic risk for PTSD. This suggests potential differences in care seeking or clinical presentation in males that may not lead to the same diagnoses. BPS also showed elevated male- and female-derived PTSD PRS, but BPSA was only significantly associated with the female-derived PTSD PRS. This may indicate that genetic loci that interact with certain types of trauma, such as military trauma exposures identified within males of the PTSD GWAS [27], may be more closely associated with suicide death risk than attempt.

Finally, the clinical evaluation of BPS versus NBPS showed a striking overrepresentation of comorbid diagnoses, particularly trauma-associated diagnoses, but polygenic risk comparison yielded no significant findings. It is possible that genetic liability among BPS and NBPS is similar, but patients diagnosed with BP may receive additional clinical evaluation leading to identification of comorbid diagnoses such as PTSD.

### Other potential risk factors

The clinically directed genetic analyses generated novel correlations of ADHD and insomnia polygenic risk in BPS versus C. ADHD diagnosis was also overrepresented in BPS versus NBPS. ADHD has been shown to be correlated with suicidal behavior [42] and may increase risk when comorbid with BP [43]. Together, ADHD and BP could be theorized to increase risk for “impulsive aggression”, a potentially important risk factor for suicidal behavior [44]. Insomnia has also been correlated with increased suicide behavior risk [45] and may be an important predictive factor for the presence of comorbid disease, such as PTSD [46]. It is notable that in comparing BPS to NBPS, 44.8% versus 18.3% of females and 28.4% versus 13.2% of males had a concurrent diagnosis of insomnia, respectively. This suggests that female BPS are more frequently diagnosed with insomnia despite a male-driven genetic finding, which may indicate sex-specific differences in diagnosis or care seeking.

### Limitations

This study had several limitations. Among these is modest sample size, though the assessed sample of BPS is the largest known sample of its kind. Also, replication is currently not possible as no comparable BPS sample is currently known to exist. Though not the focus of this study, efforts are also underway to collect larger BPSA and BPNSA samples with clinical data to allow more effective comparisons of these groups.

The use of two distinct cohorts introduced several potential limitations. Different genotyping arrays led to a limited number of overlapping variants, somewhat limiting the efficacy of imputation and PRS calculation. In addition, population ascertainment differed substantially: a general population sample (USGRS) versus an assembled research sample (NIMH), both with strengths but potentially biased comparisons. For example, the USGRS samples were not evaluated with a comprehensive diagnostic interview, potentially missing important comorbidities and weakening current associations. It must also be noted that diagnoses within a population sample arise only through individuals seeking clinical encounters and are less likely to represent every diagnosis an individual might have. This leads to a high likelihood that individuals with undiagnosed BP may be present within NBPS, potentially weakening comparisons between these groups. Conversely, the NIMH sample represents voluntary cohorts that may not adequately reflect the general population, but who were rigorously assessed by multiple providers via a consistent, extensive questionnaire to provide best estimate diagnoses. Despite this rigorous evaluation, however, all potentially relevant comorbidities, and particularly the personality disorders other than antisocial personality disorder, were not systematically evaluated as part of the core questionnaire, being identified through family informant, medical records, and early life trauma evaluation which were not available for all subjects. Indeed, it was noted that <1% of BP individuals within the NIMH cohort were diagnosed with borderline personality disorder, though a recent meta-analysis of the frequency of comorbid borderline personality disorder in bipolar disorder predicted an average of 21.6% [47], suggesting that many diagnoses may have been missed in this cohort. Finally, the evaluation of only Northern European subjects limits the generalizability of this study and was necessitated by a limited number of samples from other ethnicities. Ongoing efforts to collect a larger, more diverse, and cohesive sample are underway. Despite these inherent challenges, however, it is notable that the comparison of such datasets is necessitated by the relative rarity of these phenotypes (particularly suicide death) and is supported by evidence of a convergent finding within clinical and genetic data evaluations of prior trauma as a potential factor in suicide death risk in BP, illustrating the potential power of this complimentary approach.

## Conclusion

This study represents the first large-scale evaluation of suicide death in BP to utilize a combined clinical and genetic approach. In identifying converging evidence of factors specifically associated with suicide death, particularly prior trauma and its associated phenotypes, this study provides potentially tractable targets for future evaluation and indicates the need to specifically collect and evaluate individuals who have died from suicide to best characterize risk factors for this preventable outcome. Findings may serve to improve current screening measures for suicide death risk and, ultimately, help reduce death by suicide.

## Supplementary information

Online Supplement
