# Supplementary material for: Assessment of suicide attempt and death in bipolar affective disorder: a combined clinical and genetic approach
Source: Transl Psychiatry. 2021 Jul 7;11:379. doi: 10.1038/s41398-021-01500-w (PMC8263578; doi:10.1038/s41398-021-01500-w)
Supplement: Supplementary file 1 — Online Supplement [file 41398_2021_1500_MOESM1_ESM.docx]

Supplemental Materials

Contents

[Supplemental materials and methods: 1](#_Toc74905402)

[NIMH Sample set acknowledgements: 4](#_Toc74905403)

[Table S1: Diagnostic codes used to generate comborbid psychiatric diagnostic categories 9](#_Toc74905404)

[Table S2: Primary Clinical Diagnostic Category Analysis Results 11](#_Toc74905405)

[Table S3: Sex Specific Clinical Diagnostic Category Analysis Results 12](#_Toc74905406)

[Table S4: Utah USGRS BPS Versus NBPS Clinical Diagnostic Category Analysis Results 13](#_Toc74905407)

[Table S5: Utah USGRS Sex-Specific BPS Versus NBPS Clinical Diagnostic Category Analysis Results 14](#_Toc74905408)

[Table S6: Primary PRS Analysis Results 15](#_Toc74905409)

[Table S7: Sex-Specific PRS Analysis Results 19](#_Toc74905410)

[Table S8: Utah USGRS BPS Versus NBPS PRS Analysis Results 26](#_Toc74905411)

[Table S9: Utah USGRS BPS Versus NBPS Sex-Specific PRS Analysis Results 27](#_Toc74905412)

[References 28](#_Toc74905413)

# Supplemental materials and methods:

Additional NIMH sample collection details:

All NIMH samples were evaluated utilizing the Diagnostic interview for genetic studies ^1^. This interview included questions to systematically evaluate a number of comorbid diagnoses including anxiety disorders, eating disorders, and antisocial personality disorder (other personality disorders were not systematically evaluated). Questions also indirectly assessed prior traumatic exposure for all subjects and an additional early life trauma questionnaire was included that the majority of patients (>60%) had responded to. Finally, other diagnoses not formally assessed by questionnaire were identified by collection sites through medical records and family informant information, noting that these data were not available for all subjects.

Processing and Quality Control of genotyped samples:

All samples were screened for ancestry with principal component analyses, and subjects having >=0.90% predicted European ancestry were retained to maximize the homogeneity of the genetic samples. European samples were identified by utilizing principal components within an in-house modified version of the freely available tool, kgp2anc (<https://github.com/freeseek/kgp2anc>). Ancestry principal components were also included as covariates in all regression analyses of polygenic risk. Both the Utah and NIMH data were previously screened for genotype quality at the SNP level and the subject level, and one individual per every pair of individuals with relatedness of pi-hat ≥ 0.120 was removed with preferential removal of individuals that demonstrated relatedness to multiple samples.

The Utah and NIMH genotype data were combined across 72 215 common SNPs, representing all overlapping SNPs between the two array designs: Affymetrix Genome-Wide Human SNP Array 6.0 (<https://www.thermofisher.com/us/en/home/life-science/microarray-analysis/affymetrix.html>) versus Illumina Infinium Psycharray (<https://www.illumina.com/products/by-type/microarray-kits/infinium-psycharray.html>). Of those, 28 SNPs exhibited differential allele frequencies across datasets (p-value < 10^-6^) and were excluded. Data from 72 187 common SNPs were imputed using the Michigan Imputation Server^2^ with Haplotype Reference Consortium reference panel to a total of 39 235 157 common variants. The imputed SNPs were filtered by the imputation quality (R^2^ > 0.5, AvgCall > 0.9) and minor allele frequency (>0.001), resulting in 7 437 997 SNPs used for the analyses.

Clinical Analysis details:

Clinical comorbid psychiatric diagnoses were available for all subjects in the format of ICD codes as part of the electronic health records of USGRS samples and additionally available best-estimate diagnoses collected in the DIGS for the NIMH sample sets were compared. All clinical diagnoses found in at least two individuals from both the NIMH and USGRS populations were retained. Available diagnoses were merged into 5 diagnostic categories of related diagnoses based on consensus of M.D./Ph.D.-level clinicians (E.M., B.K., A.D.) to control for differences in preferences for particular diagnostic codes across providers and locations (see supplementary table 1 for diagnostic category contents). Subjects were then classified on a yes/no basis for having any of the comorbid diagnoses in the cluster. Individuals were excluded if they demonstrated significant missing clinical data. Specifically, subjects were excluded from the USGRS sample who did not have any linked diagnostic information available from electronic health records. Subjects from the NIMH data were excluded if they were missing suicide attempt history or comorbid clinical diagnostic history as denoted by missing fields entered in the survey for the given subject. Logistic regression models were generated on all included subjects accounting for individual age, sex, equivalent education level and completeness of medical record data. Education level data were available for all subjects as either years of education completed (NIMH data) or as categories (Utah suicide data. For consistency, education data given in years was converted to categories as follows: 0-8 years of reported education were classified as “8^th^ grade or lower”, 9-11 years of education were classified as “9^th^ to 12^th^, no graduation”, 12 years as “HS grad/GED”, 13 as “some college, no degree,” 14 years as “associates degree or equivalent,” 15-17 years as “Bachelors or equivalent,” 18-20 years as “Masters or equivalent,” and 21+ years as “PhD or equivalent.” ). It is noted that several individuals in the NIMH data also had self-reported answers for degree or education level reached in addition to years of education and these data were used to validate that categorical conversion of years of education was well-representative of actual attainment. Completeness of medical record data was incorporated into a categorical variable where individuals were assigned to a low, medium, or high group based on the number of available clinical records per subject. Each medical record category represented approximately 1/3 of the total group of individuals. All assessments tested the rate of comorbid clinical categories between comparison groups.

Genetic analysis details:

This assessment focused on utilizing the extensive genetic data we have available for all study subjects to generate potentially relevant genetic profiles for these population groups. Profiles were generated through the calculation of polygenic risk scores (PRS) which are essentially weighted sum scores, where a score for an individual in the current study is calculated by the summation of each SNP multiplied by the effect size of that SNP from an external discovery GWAS. PRS to be calculated were selected based on significant results from the clinical analyses. These PRS were calculated from publicly available summary statistics for ADHD^3,4^, anxiety^5^, insomnia^6^, PTSD^7^, suicide attempt^8^, and neuroticism.^9^ Many of these studies included sub phenotypes and/or sex-specific analyses (male- and female-derived polygenic risk marker sets). All such subgroups were included in all analyses, with the exception that sex-specific analyses were confined to matching sex-derived polygenic markers. Sex-derived PRS groups were included within the all-subject analyses, however, hypothesizing that specific sex-derived markers might be informative for interrogating differences in comparison groups where known sex-specific differences exist, such as suicide attempt versus suicide death.

PRS were calculated using PRSice 2.0^10^ with the default pruning and thresholding parameters and the p-value threshold of 1. This threshold was decided upon in the absence of any prior analyses of this type in the literature. In addition, PRS for suicide death from USGRS data (utilizing a cross-validation procedure) was included as fully described elsewhere ^11^. Finally, a suicide attempt PRS specifically within bipolar disorder subjects was generated from published psychiatric genetics consortium data ^8^ with summary statistics excluding the samples from this testing cohort, which specifically included the GAIN and TGEN study populations.

Statistics and image preparation:

All statistical analyses were performed in R ^12^. Clinical forest plots were prepared via the R package “forestplot” ^13^. Boxplots for PRS results were generated utilizing the R package “ggplot2” ^14^.

# NIMH Sample set acknowledgements:

**Bipolar samples:**

Genome-wide SNP genotyping of the NIMH samples was performed through the Genetic Association Information Network under the direction of The Bipolar Genome Study (BiGS) Consortium. The Principal Investigators and Co-Investigators were: University of California San Diego, La Jolla, CA, John R. Kelsoe, M.D. (PI), Tiffany A. Greenwood, Ph.D., Thomas B. Barrett, M.D., Ph.D., Caroline M. Nievergelt, Ph.D., Rebecca McKinney, Paul D. Shilling, Ph.D.; Scripps Research Institute, La Jolla, CA: Nicholas Schork, Ph.D. (PI), Erin N. Smith, Ph.D., Cinnamon S. Bloss, Ph.D.; Indiana University, Bloomington, IN, John I. Nurnberger, Jr., M.D. (PI), Howard J. Edenberg, Ph.D., Tatiana Foroud, Ph.D., Daniel M. Koller; University of Chicago, Chicago, IL, Elliot Gershon, M.D. (PI), Chunyu Liu, Ph.D., Judith A. Badner, Ph.D.; Rush University Medical Center, Chicago, IL, William A. Scheftner, M.D.; Howard University, Washington, DC, William B. Lawson, M.D. (PI), Evaristus A. Nwulia, M.D., Maria Hipolito, M.D.; University of Iowa, Iowa City, IA, William Coryell, M.D. (PI); Washington University, St. Louis, MO, John Rice, Ph.D. (PI); University of California San Francisco, San Francisco, CA, William Byerley, M.D. (PI); National Institute of Mental Health, Bethesda, MD, Francis McMahon, M.D. (PI), Thomas G. Schulze, M.D.; University of Pennsylvania, Philadelphia, PA, Wade Berrettini, M.D., Ph.D. (PI); Johns Hopkins University, Baltimore, MD, James B. Potash, M.D. (PI), Peter P. Zandi, Ph.D., Pamela B. Mahon, Ph.D.; University of Michigan, Ann Arbor, MI, Melvin G. McInnis, M.D. (PI), Sebastian Zöllner, Ph.D., Peng Zhang; The Translational Genomics Research Institute, Phoenix, AZ, David Craig, Ph.D. (PI), Szabolics Szelinger.

Data and biomaterials were collected in four projects that participated in the National Institute of Mental Health (NIMH) Bipolar Disorder Genetics Initiative. From 1991-98, the Principal Investigators and Co-Investigators were: Indiana University, Indianapolis, IN, U01 MH46282, John Nurnberger, M.D., Ph.D., Marvin Miller, M.D., and Elizabeth Bowman, M.D.; Washington University, St. Louis, MO, U01 MH46280, Theodore Reich, M.D., Allison Goate, Ph.D., and John Rice, Ph.D.; Johns Hopkins University, Baltimore, MD U01 MH46274, J. Raymond DePaulo, Jr., M.D., Sylvia Simpson, M.D., MPH, and Colin Stine, Ph.D.; NIMH Intramural Research Program, Clinical Neurogenetics Branch, Bethesda, MD, Elliot Gershon, M.D., Diane Kazuba, B.A., and Elizabeth Maxwell, M.S.W.

Data and biomaterials were collected as part of ten projects that participated in the National Institute of Mental Health (NIMH) Bipolar Disorder Genetics Initiative. From 1999-03, the Principal Investigators and Co-Investigators were: Indiana University, Indianapolis, IN, R01 MH59545, John Nurnberger, M.D., Ph.D., Marvin J. Miller, M.D., Elizabeth S. Bowman, M.D., N. Leela Rau, M.D., P. Ryan Moe, M.D., Nalini Samavedy, M.D., Rif El-Mallakh, M.D. (at University of Louisville), Husseini Manji, M.D. (at Wayne State University), Debra A. Glitz, M.D. (at Wayne State University), Eric T. Meyer, M.S., Carrie Smiley, R.N., Tatiana Foroud, Ph.D., Leah Flury, M.S., Danielle M. Dick, Ph.D., Howard Edenberg, Ph.D.; Washington University, St. Louis, MO, R01 MH059534, John Rice, Ph.D., Theodore Reich, M.D., Allison Goate, Ph.D., Laura Bierut, M.D. ; Johns Hopkins University, Baltimore, MD, R01 MH59533, Melvin McInnis M.D. , J. Raymond DePaulo, Jr., M.D., Dean F. MacKinnon, M.D., Francis M. Mondimore, M.D., James B. Potash, M.D., Peter P. Zandi, Ph.D., Dimitrios Avramopoulos M.D., Ph.D., and Jennifer Payne, M.D.; University of Pennsylvania, PA, R01 MH59553, Wade Berrettini M.D., Ph.D.; University of California at Irvine, CA, R01 MH60068, William Byerley M.D., and Mark Vawter M.D.; University of Iowa, IA, R01 MH059548, William Coryell M.D. , and Raymond Crowe M.D. ; University of Chicago, IL, R01 MH59535, Elliot Gershon, M.D., Judith Badner Ph.D. , Francis McMahon M.D. , Chunyu Liu Ph.D., Alan Sanders M.D., Maria Caserta, Steven Dinwiddie M.D., Tu Nguyen, Donna Harakal; University of California at San Diego, CA, R01 MH59567, John Kelsoe, M.D., Rebecca McKinney, B.A.; Rush University, IL, R01 MH059556, William Scheftner M.D. , Howard M. Kravitz, D.O., M.P.H., Diana Marta, B.S., Annette Vaughn-Brown, MSN, RN, and Laurie Bederow, MA; NIMH Intramural Research Program, Bethesda, MD, 1Z01MH002810-01, Francis J. McMahon, M.D., Layla Kassem, PsyD, Sevilla Detera-Wadleigh, Ph.D., Lisa Austin, Ph.D., Dennis L. Murphy, M.D.

Data and biomaterials were collected as part of eleven projects (Study 40) that participated in the National Institute of Mental Health (NIMH) Bipolar Disorder Genetics Initiative. From 2003-2007, the Principal Investigators and Co-Investigators were: Indiana University, Indianapolis, IN, R01 MH59545, John Nurnberger, M.D., Ph.D., Marvin J. Miller, M.D., Elizabeth S. Bowman, M.D., N. Leela Rau, M.D., P. Ryan Moe, M.D., Nalini Samavedy, M.D., Rif El-Mallakh, M.D. (at University of Louisville), Husseini Manji, M.D. (at Johnson and Johnson), Debra A. Glitz, M.D. (at Wayne State University), Eric T. Meyer, Ph.D., M.S. (at Oxford University, UK), Carrie Smiley, R.N., Tatiana Foroud, Ph.D., Leah Flury, M.S., Danielle M. Dick, Ph.D (at Virginia Commonwealth University), Howard Edenberg, Ph.D.; Washington University, St. Louis, MO, R01 MH059534, John Rice, Ph.D., Theodore Reich, M.D., Allison Goate, Ph.D., Laura Bierut, M.D. K02 DA21237; Johns Hopkins University, Baltimore, M.D., R01 MH59533, Melvin McInnis, M.D., J. Raymond DePaulo, Jr., M.D., Dean F. MacKinnon, M.D., Francis M. Mondimore, M.D., James B. Potash, M.D., Peter P. Zandi, Ph.D., Dimitrios Avramopoulos M.D.,Ph.D., and Jennifer Payne, M.D.; University of Pennsylvania, PA, R01 MH59553, Wade Berrettini, M.D., Ph.D.; University of California at San Francisco, CA, R01 MH60068, William Byerley, M.D., and Sophia Vinogradov, M.D.; University of Iowa, IA, R01 MH059548, William Coryell, M.D., and Raymond Crowe, M.D.; University of Chicago, IL, R01 MH59535, Elliot Gershon, M.D., Judith Badner, Ph.D., Francis McMahon, M.D., Chunyu Liu, Ph.D., Alan Sanders, M.D., Maria Caserta, Steven Dinwiddie, M.D., Tu Nguyen, Donna Harakal; University of California at San Diego, CA, R01 MH59567, John Kelsoe, M.D., Rebecca McKinney, B.A.; Rush University, IL, R01 MH059556, William Scheftner, M.D., Howard M. Kravitz, D.O., M.P.H., Diana Marta, B.S., Annette Vaughn-Brown, M.S.N., R.N., and Laurie Bederow, M.A.; NIMH Intramural Research Program, Bethesda, MD, 1Z01MH002810-01, Francis J. McMahon, M.D., Layla Kassem, Psy.D., Sevilla Detera-Wadleigh, Ph.D., Lisa Austin, Ph.D., Dennis L. Murphy, M.D.; Howard University, William B. Lawson, M.D., Ph.D., Evarista Nwulia, M.D., and Maria Hipolito, M.D.

**Controls:**

Control subjects from the National Institute of Mental Health Schizophrenia Genetics Initiative (NIMH-GI), data and biomaterials are being collected by the "Molecular Genetics of Schizophrenia II" (MGS-2) collaboration. The investigators and co-investigators are: ENH/Northwestern University, Evanston, IL, MH059571, Pablo V. Gejman, M.D. (Collaboration Coordinator; PI), Alan R. Sanders, M.D.; Emory University School of Medicine, Atlanta, GA, MH59587, Farooq Amin, M.D. (PI); Louisiana State University Health Sciences Center; New Orleans, Louisiana, MH067257, Nancy Buccola APRN, B.C., M.S.N. (PI); University of California-Irvine, Irvine, CA, MH60870, William Byerley, M.D. (PI); Washington University, St. Louis, MO, U01, MH060879, C. Robert Cloninger, M.D. (PI); University of Iowa, Iowa, IA, MH59566, Raymond Crowe, M.D. (PI), Donald Black, M.D.; University of Colorado, Denver, CO, MH059565, Robert Freedman, M.D. (PI); University of Pennsylvania, Philadelphia, PA, MH061675, Douglas Levinson, M.D. (PI); University of Queensland, Queensland, Australia, MH059588, Bryan Mowry, M.D. (PI); Mt. Sinai School of Medicine, New York, NY, MH59586, Jeremy Silverman, Ph.D. (PI).

In addition, cord blood samples were collected by Vishwajit Nimgaonkar's group at the University of Pittsburgh, as part of a multi-institutional collaborative research project with Jordan Smoller, M.D., D.Sc., and Pamela Sklar, M.D., Ph.D., Massachusetts General Hospital (grant MH 63420).

| Table S1: Diagnostic codes used to generate comborbid psychiatric diagnostic categories | | | | | |
| --- | --- | --- | --- | --- | --- |
| Category | ICD_Version | | | Code | Description |
| Non-Traumatic Anxiety Disorders | | | ICD-9 | 300.00 | Anxiety State |
| Non-Traumatic Anxiety Disorders | | | ICD-9 | 300.01 | Panic disorder without agorophbia |
| Non-Traumatic Anxiety Disorders | | | ICD-9 | 300.02 | Generalized Anxiety Disorder |
| Non-Traumatic Anxiety Disorders | | | ICD-9 | 300.09 | Other Anxiety States |
| Non-Traumatic Anxiety Disorders | | | ICD-9 | 300.11 | Conversion disorder |
| Non-Traumatic Anxiety Disorders | | | ICD-9 | 300.21 | Agoraphobia with Panic Disorder |
| Non-Traumatic Anxiety Disorders | | | ICD-9 | 300.22 | Agoraphobia without mention of panic attacks |
| Non-Traumatic Anxiety Disorders | | | ICD-9 | 300.23 | Social Phobia |
| Non-Traumatic Anxiety Disorders | | | ICD-9 | 300.29 | Other isolated phobias |
| Non-Traumatic Anxiety Disorders | | | ICD-9 | 300.70 | Hypochondriasis |
| Non-Traumatic Anxiety Disorders | | | ICD-9 | 300.82 | Undifferentiated somatoform Disorder |
| Non-Traumatic Anxiety Disorders | | | ICD-10 | F40.00 | Agorophobia |
| Non-Traumatic Anxiety Disorders | | | ICD-10 | F40.01 | Agorophobia with panic disorder |
| Non-Traumatic Anxiety Disorders | | | ICD-10 | F40.02 | Agorophobia without panic disorder |
| Non-Traumatic Anxiety Disorders | | | ICD-10 | F40.10 | Social Phobia, unspecified |
| Non-Traumatic Anxiety Disorders | | | ICD-10 | F40.11 | Social Phobia, generalized |
| Non-Traumatic Anxiety Disorders | | | ICD-10 | F41.0 | Panic Disorder |
| Non-Traumatic Anxiety Disorders | | | ICD-10 | F41.1 | Generalized Anxiety Disorder |
| Non-Traumatic Anxiety Disorders | | | ICD-10 | F41.9 | Anxiety disorder, unspecified |
| Non-Traumatic Anxiety Disorders | | | ICD-10 | F44.4 | Conversion disorder, motor symptom |
| Non-Traumatic Anxiety Disorders | | | ICD-10 | F44.5 | Conversion disorder, seizures/convulsions |
| Non-Traumatic Anxiety Disorders | | | ICD-10 | F44.6 | Conversion disorder, sensory |
| Non-Traumatic Anxiety Disorders | | | ICD-10 | F44.7 | Conversion disorder, mixed |
| Non-Traumatic Anxiety Disorders | | | ICD-10 | F45.21 | Hypochondriasis |
| Behavioral Disorders | | | ICD-9 | 312.80 | Conduct disorder, unclassified |
| Behavioral Disorders | | | ICD-9 | 312.81 | Conduct disorder, childhood onset |
| Behavioral Disorders | | | ICD-9 | 312.82 | Conduct disorder, adolescent onset |
| Behavioral Disorders | | | ICD-9 | 312.90 | Unspecified disturbance of conduct |
| Behavioral Disorders | | | ICD-9 | 313.81 | Oppositional defiant disorder |
| Behavioral Disorders | | | ICD-9 | 314.00 | Attention deficit disorder of childhood |
| Behavioral Disorders | | | ICD-9 | 314.01 | Attention deficit disorder with hyperactivity |
| Behavioral Disorders | | | ICD-9 | 314.10 | Hyperkinesis with developmental delay |
| Behavioral Disorders | | | ICD-9 | 314.20 | Hyperkinetic conduct disorder |
| Behavioral Disorders | | | ICD-9 | 314.80 | Other manifistations of hyperkinetic syndrome |
| Behavioral Disorders | | | ICD-9 | 314.90 | Unspecified hyperkinetic syndrome |
| Behavioral Disorders | | | ICD-10 | F90.0 | Attention deficit hyperactivity disorder, inattentive type |
| Behavioral Disorders | | | ICD-10 | F90.1 | Attention deficit hyperactivity disorder, hyperactive type |
| Behavioral Disorders | | | ICD-10 | F90.2 | Attention deficit hyperactivity disorder , combined type |
| Behavioral Disorders | | | ICD-10 | F90.8 | Attention deficit hyperactivity disorder, other type |
| Behavioral Disorders | | | ICD-10 | F90.9 | Attention deficit hyperactivity disorder, unspecified type |
| Behavioral Disorders | | | ICD-10 | F91.0 | Conduct disorder, confined to family context |
| Behavioral Disorders | | | ICD-10 | F91.1 | Conduct disorder, childhood onset |
| Behavioral Disorders | | | ICD-10 | F91.2 | Conduct disorder, adolescent onset |
| Behavioral Disorders | | | ICD-10 | F91.3 | Oppositional defiant disorder |
| Category | | ICD_Version | | Code | Description |
| Behavioral Disorders | ICD-10 | | | F91.8 | Other conduct disorder |
| Behavioral Disorders | | | ICD-10 | F91.9 | Conduct disorder, unspecified |
| Personality Disorders | | | ICD-9 | 301.22 | Schizotypal personality disorder |
| Personality Disorders | | | ICD-9 | 301.50 | Histrionic personality disorder |
| Personality Disorders | | | ICD-9 | 301.70 | Antisocial personality disorder |
| Personality Disorders | | | ICD-9 | 301.80 | Other personality disorder |
| Personality Disorders | | | ICD-9 | 301.81 | Narcissitic personality disorder |
| Personality Disorders | | | ICD-9 | 301.82 | Avoidant personality disorder |
| Personality Disorders | | | ICD-9 | 301.83 | Borderline personality disorder |
| Personality Disorders | | | ICD-9 | 301.9 | Unspecified personality disorder |
| Personality Disorders | | | ICD-10 | F60.2 | Antisocial personality disorder |
| Personality Disorders | | | ICD-10 | F60.3 | Borderline personality disorder |
| Personality Disorders | | | ICD-10 | F60.4 | Histrionic personality disorder |
| Personality Disorders | | | ICD-10 | F60.6 | Avoidant personality disorder |
| Personality Disorders | | | ICD-10 | F60.80 | Other specific personality disorder |
| Personality Disorders | | | ICD-10 | F60.81 | Narcissitic personality disorder |
| Personality Disorders | | | ICD-10 | F60.89 | Other specific personality disorder |
| Personality Disorders | | | ICD-10 | F60.9 | Personality disorder, unspecified |
| Eating Disorders | | | ICD-9 | 307.10 | Anorexia Nervosa |
| Eating Disorders | | | ICD-9 | 307.50 | Other unspecified eating disorder |
| Eating Disorders | | | ICD-9 | 307.51 | Bulimia nervosa |
| Eating Disorders | | | ICD-10 | F50.00 | Anorexia Nervosa, unspecified |
| Eating Disorders | | | ICD-10 | F50.01 | Anorexia Nervosa, restricting type |
| Eating Disorders | | | ICD-10 | F50.02 | Anorexia Nervosa, binge eating/purging type |
| Eating Disorders | | | ICD-10 | F50.2 | Bulimia nervosa |
| Eating Disorders | | | ICD-10 | F50.8 | Other eating disorders |
| Eating Disorders | | | ICD-10 | F50.9 | Eating Disorder, unspecified |
| PTSD | | | ICD-9 | 309.81 | Post-traumatic stress disorder |
| PTSD | | | ICD-10 | F43.10 | Post-traumatic stress disorder, unspecified |
| PTSD | | | ICD-10 | F43.11 | Post-traumatic stress disorder, acute |
| PTSD | | | ICD-10 | F43.12 | Post-traumatic stress disorder, chronic |

| Table S2: Primary Clinical Diagnostic Category Analysis Results | | | | | | | | | | | | | | |
| --- | --- | --- | --- | --- | --- | --- | --- | --- | --- | --- | --- | --- | --- | --- |
| SexGrp | | Diagnostic_Category | Group_1 | Group_2 | G1N | G1% | G2N | G2% | Beta | OR | OR95L | | OR95U | Corrected_P |
| All | | Pers_DO | BPS | BPSA | 97 | 27.5% | 66 | 8.3% | 1.52 | 4.57 | 3.00 | | 6.96 | 2.18E-11 |
| All | | PTSD | BPS | BPSA | 77 | 21.8% | 50 | 6.3% | 1.58 | 4.86 | 3.09 | | 7.64 | 5.95E-11 |
| All | | Pers_DO | BPS | BPNSA | 97 | 27.5% | 45 | 5.5% | 1.41 | 4.11 | 2.61 | | 6.49 | 6.00E-09 |
| All | | Eating_DO | BPS | BPSA | 13 | 3.7% | 144 | 18.0% | -1.62 | 0.20 | 0.10 | | 0.37 | 2.19E-06 |
| All | | PTSD | BPS | BPNSA | 77 | 21.8% | 59 | 7.2% | 1.04 | 2.82 | 1.81 | | 4.39 | 1.37E-05 |
| All | | Non_Trauma_Anx_DO | BPS | BPNSA | 236 | 66.9% | 387 | 47.0% | 0.77 | 2.17 | 1.55 | | 3.03 | 1.39E-05 |
| All | | Non_Trauma_Anx_DO | BPS | BPSA | 236 | 66.9% | 446 | 55.8% | 0.68 | 1.98 | 1.42 | | 2.77 | 1.26E-04 |
| All | | Eating_DO | BPS | BPNSA | 13 | 3.7% | 89 | 10.8% | -1.31 | 0.27 | 0.13 | | 0.54 | 3.88E-04 |
| All | | PTSD | BPSA | BPNSA | 50 | 6.3% | 59 | 7.2% | -0.42 | 0.66 | 0.44 | | 0.99 | 7.47E-02 |
| All | | Eating_DO | BPSA | BPNSA | 144 | 18.0% | 89 | 10.8% | 0.21 | 1.23 | 0.90 | | 1.69 | 2.89E-01 |
| All | | Behavioral_DO | BPS | BPSA | 93 | 26.3% | 196 | 24.5% | -0.21 | 0.81 | 0.57 | | 1.14 | 3.10E-01 |
| All | | Behavioral_DO | BPSA | BPNSA | 196 | 24.5% | 146 | 17.7% | 0.16 | 1.17 | 0.90 | | 1.52 | 3.11E-01 |
| All | | Pers_DO | BPSA | BPNSA | 66 | 8.3% | 45 | 5.5% | 0.11 | 1.12 | 0.73 | | 1.71 | 6.95E-01 |
| All | | Non_Trauma_Anx_DO | BPSA | BPNSA | 446 | 55.8% | 387 | 47.0% | 0.02 | 1.03 | 0.82 | | 1.27 | 8.82E-01 |
| All | | Behavioral_DO | BPS | BPNSA | 93 | 26.3% | 146 | 17.7% | -0.03 | 0.97 | 0.68 | | 1.39 | 8.69E-01 |
| Group key: | | | | | | | | | | | |  |  |  |
| BPS | individuals with bipolar disorder who died by suicide | | | | | | | | | | |  |  |  |
| BPSA | individuals with bipolar disorder who have a history of one or more suicide attempts | | | | | | | | | | |  |  |  |
| BPNSA | individuals with bipolar disorder who have no history of a suicide attempt | | | | | | | | | | |  |  |  |
| Note: Significant results after corrections are highlighted in blue | | | | | | | | | | | |  |  |  |

| Table S3: Sex Specific Clinical Diagnostic Category Analysis Results | | | | | | | | | | | | | | |  |
| --- | --- | --- | --- | --- | --- | --- | --- | --- | --- | --- | --- | --- | --- | --- | --- |
| SexGrp | Diagnostic_Category | Group_1 | Group_2 | G1N | G1% | | G2N | | G2% | Beta | OR | OR95L | OR95U | Corrected_P |  |
| Female | Pers_DO | BPS | BPSA | 58 | 43.3% | | 36 | | 6.7% | 2.27 | 9.72 | 5.45 | 17.37 | 4.47E-13 |  |
| Female | PTSD | BPS | BPSA | 51 | 38.1% | | 34 | | 6.3% | 2.10 | 8.18 | 4.67 | 14.35 | 3.35E-12 |  |
| Female | Pers_DO | BPS | BPNSA | 58 | 43.3% | | 13 | | 3.0% | 2.47 | 11.8 | 5.40 | 25.83 | 6.33E-09 |  |
| Female | PTSD | BPS | BPNSA | 51 | 38.1% | | 29 | | 6.7% | 1.78 | 5.92 | 3.11 | 11.29 | 4.86E-07 |  |
| Female | Eating_DO | BPS | BPSA | 12 | 9.0% | | 127 | | 23.6% | -1.70 | 0.18 | 0.09 | 0.37 | 1.13E-05 |  |
| Female | Non_Trauma_Anx_DO | BPS | BPNSA | 117 | 87.3% | | 243 | | 56.1% | 1.24 | 3.47 | 1.84 | 6.55 | 6.15E-04 |  |
| Female | Non_Trauma_Anx_DO | BPS | BPSA | 117 | 87.3% | | 328 | | 61.1% | 1.15 | 3.15 | 1.74 | 5.69 | 6.13E-04 |  |
| Male | Non_Trauma_Anx_DO | BPS | BPNSA | 119 | 54.3% | | 144 | | 36.8% | 0.68 | 1.96 | 1.30 | 2.98 | 5.50E-03 |  |
| Male | Eating_DO | BPS | BPNSA | 1 | 0.5% | | 15 | | 3.8% | -2.91 | 0.05 | 0.01 | 0.47 | 2.67E-02 |  |
| Male | Eating_DO | BPS | BPSA | 1 | 0.5% | | 17 | | 6.5% | -2.68 | 0.07 | 0.01 | 0.55 | 3.42E-02 |  |
| Female | Eating_DO | BPS | BPNSA | 12 | 9.0% | | 74 | | 17.1% | -0.99 | 0.37 | 0.17 | 0.82 | 3.70E-02 |  |
| Male | Non_Trauma_Anx_DO | BPS | BPSA | 119 | 54.3% | | 118 | | 45.0% | 0.53 | 1.70 | 1.09 | 2.63 | 4.54E-02 |  |
| Male | PTSD | BPSA | BPNSA | 16 | 6.1% | | 30 | | 7.7% | -0.55 | 0.57 | 0.30 | 1.11 | 2.32E-01 |  |
| Male | Pers_DO | BPS | BPSA | 39 | 17.8% | | 30 | | 11.5% | 0.48 | 1.62 | 0.88 | 2.98 | 2.64E-01 |  |
| Female | Behavioral_DO | BPS | BPSA | 33 | 24.6% | | 123 | | 22.9% | -0.33 | 0.72 | 0.44 | 1.19 | 4.04E-01 |  |
| Male | PTSD | BPS | BPSA | 26 | 11.9% | | 16 | | 6.1% | 0.43 | 1.54 | 0.75 | 3.18 | 4.55E-01 |  |
| Female | PTSD | BPSA | BPNSA | 34 | 6.3% | | 29 | | 6.7% | -0.31 | 0.74 | 0.43 | 1.26 | 4.70E-01 |  |
| Female | Eating_DO | BPSA | BPNSA | 127 | 23.6% | | 74 | | 17.1% | 0.20 | 1.22 | 0.85 | 1.73 | 4.60E-01 |  |
| Female | Behavioral_DO | BPSA | BPNSA | 123 | 22.9% | | 66 | | 15.2% | 0.19 | 1.21 | 0.85 | 1.72 | 4.64E-01 |  |
| Male | Pers_DO | BPS | BPNSA | 39 | 17.8% | | 32 | | 8.2% | 0.32 | 1.38 | 0.75 | 2.52 | 4.45E-01 |  |
| Male | Eating_DO | BPSA | BPNSA | 17 | 6.5% | | 15 | | 3.8% | 0.35 | 1.42 | 0.67 | 2.98 | 5.12E-01 |  |
| Male | Non_Trauma_Anx_DO | BPSA | BPNSA | 118 | 45.0% | | 144 | | 36.8% | 0.15 | 1.16 | 0.82 | 1.65 | 5.47E-01 |  |
| Female | Pers_DO | BPSA | BPNSA | 36 | 6.7% | | 13 | | 3.0% | 0.28 | 1.32 | 0.67 | 2.60 | 5.58E-01 |  |
| Male | Behavioral_DO | BPSA | BPNSA | 73 | 27.9% | | 80 | | 20.5% | 0.11 | 1.11 | 0.74 | 1.66 | 7.59E-01 |  |
| Female | Behavioral_DO | BPS | BPNSA | 33 | 24.6% | | 66 | | 15.2% | -0.15 | 0.86 | 0.48 | 1.55 | 7.36E-01 |  |
| Male | Behavioral_DO | BPS | BPNSA | 60 | 27.4% | | 80 | | 20.5% | 0.12 | 1.13 | 0.70 | 1.81 | 7.15E-01 |  |
| Male | PTSD | BPS | BPNSA | 26 | 11.9% | | 30 | | 7.7% | 0.17 | 1.18 | 0.61 | 2.31 | 6.89E-01 |  |
| Male | Behavioral_DO | BPS | BPSA | 60 | 27.4% | | 73 | | 27.9% | -0.10 | 0.91 | 0.56 | 1.47 | 7.37E-01 |  |
| Female | Non_Trauma_Anx_DO | BPSA | BPNSA | 328 | 61.1% | | 243 | | 56.1% | -0.04 | 0.96 | 0.72 | 1.28 | 8.13E-01 |  |
| Male | Pers_DO | BPSA | BPNSA | 30 | 11.5% | | 32 | | 8.2% | -0.07 | 0.94 | 0.53 | 1.66 | 8.24E-01 |  |
| Group key | | | | | | | | | | | | | | |  |
| BPS | individuals with bipolar disorder who died by suicide | | | | | BPSA | | individuals with bipolar disorder who have a history of one or more suicide attempts | | | | | | | |
| BPNSA | individuals with bipolar disorder who have no prior suicide attempts | | | | | Note: Significant results after corrections are highlighted in blue | | | | | | | | |  |

| Table S4: Utah USGRS BPS Versus NBPS Clinical Diagnostic Category Analysis Results | | | | | | | | | | | | |
| --- | --- | --- | --- | --- | --- | --- | --- | --- | --- | --- | --- | --- |
| Sex_Grp | Diagnostic_Category | Group_1 | Group_2 | G1N | G1% | G2N | G2% | Beta | OR | OR95L | OR95U | Corrected_P |
| All | Pers_DO | BPS | NBPS | 97 | 27.5% | 110 | 4.4% | 1.28 | 3.61 | 2.56 | 5.08 | 9.10E-13 |
| All | Behavioral_DO | BPS | NBPS | 93 | 26.3% | 156 | 6.2% | 1.14 | 3.14 | 2.27 | 4.34 | 1.33E-11 |
| All | PTSD | BPS | NBPS | 77 | 21.8% | 106 | 4.2% | 0.97 | 2.63 | 1.83 | 3.78 | 2.85E-07 |
| All | Non_Trauma_Anx_DO | BPS | NBPS | 236 | 66.9% | 795 | 31.8% | 0.75 | 2.12 | 1.57 | 2.86 | 1.21E-06 |
| All | Eating_DO | BPS | NBPS | 13 | 3.7% | 11 | 0.4% | 1.07 | 2.91 | 1.17 | 7.25 | 2.20E-02 |
| Group key | |  |  |  |  |  |  |  |  |  |  |  |
| BPS | individuals with bipolar disorder who died by suicide | | | | | | | | | | | |
| NBPS | individuals without a diagnosis of bipolar disorder who died from suicide | | | | | | | | | | | |
| Note: Significant results after corrections are highlighted in blue | | | | | | | | | | | | |

| Table S5: Utah USGRS Sex-Specific BPS Versus NBPS Clinical Diagnostic Category Analysis Results | | | | | | | | | | | | |
| --- | --- | --- | --- | --- | --- | --- | --- | --- | --- | --- | --- | --- |
| Sex_Grp | Diagnostic_Category | Group_1 | Group_2 | G1N | G1% | G2N | G2% | Beta | OR | OR95L | OR95U | Corrected_P |
| Male | Behavioral_DO | BPS | NBPS | 60 | 27.4% | 134 | 6.7% | 1.15 | 3.17 | 2.16 | 4.67 | 4.52E-08 |
| Female | Pers_DO | BPS | NBPS | 58 | 43.3% | 35 | 6.8% | 1.50 | 4.46 | 2.58 | 7.73 | 4.78E-07 |
| Female | Non_Trauma_Anx_DO | BPS | NBPS | 117 | 87.3% | 222 | 43.4% | 1.39 | 4.00 | 2.13 | 7.51 | 5.43E-05 |
| Male | Pers_DO | BPS | NBPS | 39 | 17.8% | 75 | 3.8% | 1.00 | 2.72 | 1.71 | 4.31 | 5.60E-05 |
| Female | Behavioral_DO | BPS | NBPS | 33 | 24.6% | 22 | 4.3% | 1.36 | 3.89 | 1.96 | 7.72 | 2.06E-04 |
| Male | PTSD | BPS | NBPS | 26 | 11.9% | 57 | 2.9% | 0.96 | 2.62 | 1.54 | 4.46 | 6.25E-04 |
| Female | PTSD | BPS | NBPS | 51 | 38.1% | 49 | 9.6% | 0.92 | 2.50 | 1.47 | 4.27 | 1.05E-03 |
| Male | Non_Trauma_Anx_DO | BPS | NBPS | 119 | 54.3% | 573 | 28.8% | 0.51 | 1.66 | 1.16 | 2.37 | 6.95E-03 |
| Female | Eating_DO | BPS | NBPS | 12 | 9.0% | 8 | 1.6% | 1.56 | 4.74 | 1.39 | 16.23 | 1.47E-02 |
| Male | Eating_DO | BPS | NBPS | 1 | 0.5% | 3 | 0.2% | 0.13 | 1.13 | 0.10 | 12.83 | 9.19E-01 |
| Group key | | | | | |  |  |  |  |  |  |  |
| BPS | individuals with bipolar disorder who died by suicide | | | | | | | | | | | |
| NBPS | individuals without a diagnosis of bipolar disorder who died from suicide | | | | | | | | | | | |
| Note: Significant results after corrections are highlighted in blue | | | | | | | | | | | | |

| Table S6: Primary PRS Analysis Results | | | | | | | | |
| --- | --- | --- | --- | --- | --- | --- | --- | --- |
| SexGrp | PRS_Set | Group1 | Group2 | Beta | OR | OR95L | OR95U | Corrected_P |
| All | All_Suicide_Death_1.00 | BPS | BPNSA | 0.53 | 1.69 | 1.48 | 1.94 | 2.03E-12 |
| All | All_Suicide_Death_1.00 | BPS | C | 0.51 | 1.66 | 1.46 | 1.89 | 1.27E-12 |
| All | All_Suicide_Attempt_in_BP_no_NIMH_1.00 | BPSA | BPNSA | 0.38 | 1.47 | 1.32 | 1.63 | 1.08E-11 |
| All | All_Suicide_Death_1.00 | BPS | BPSA | 0.48 | 1.61 | 1.40 | 1.86 | 7.84E-10 |
| All | All_Suicide_Attempt_in_BP_no_NIMH_1.00 | BPNSA | C | -0.31 | 0.73 | 0.66 | 0.81 | 4.17E-08 |
| All | All_Suicide_Attempt_in_BP_no_NIMH_1.00 | BPS | BPNSA | 0.41 | 1.50 | 1.29 | 1.75 | 1.94E-06 |
| All | All_PTSD_Female_Derived_1.00 | BPSA | C | 0.19 | 1.21 | 1.09 | 1.33 | 3.62E-03 |
| All | All_Suicide_attempt_in_MDD_1.00 | BPS | BPNSA | 0.34 | 1.40 | 1.17 | 1.68 | 3.68E-03 |
| All | All_Suicide_attempt_in_MDD_1.00 | BPS | C | 0.31 | 1.36 | 1.15 | 1.62 | 4.64E-03 |
| All | All_Suicide_Attempt_in_BP_no_NIMH_1.00 | BPSA | C | 0.17 | 1.19 | 1.08 | 1.31 | 4.26E-03 |
| All | All_Worry_Subcluster_1.00 | BPSA | C | 0.17 | 1.18 | 1.08 | 1.30 | 6.04E-03 |
| All | All_PTSD_Male_Derived_1.00 | BPS | C | 0.26 | 1.30 | 1.12 | 1.52 | 8.04E-03 |
| All | All_PTSD_1.00 | BPS | C | 0.26 | 1.29 | 1.11 | 1.50 | 7.76E-03 |
| All | All_Anxiety_1.00 | BPNSA | C | 0.14 | 1.15 | 1.05 | 1.25 | 1.66E-02 |
| All | All_Suicide_attempt_in_MDD_1.00 | BPS | BPSA | 0.28 | 1.32 | 1.10 | 1.58 | 1.79E-02 |
| All | All_Anxiety_1.00 | BPSA | C | 0.14 | 1.15 | 1.05 | 1.25 | 1.81E-02 |
| All | All_ADHD_Female_Derived_1.00 | BPS | C | 0.21 | 1.23 | 1.07 | 1.41 | 1.98E-02 |
| All | All_PTSD_Female_Derived_1.00 | BPS | C | 0.20 | 1.22 | 1.07 | 1.40 | 2.53E-02 |
| All | All_ADHD_All_1.00 | BPSA | C | 0.13 | 1.13 | 1.03 | 1.25 | 5.21E-02 |
| All | All_PTSD_Female_Derived_1.00 | BPSA | BPNSA | 0.14 | 1.15 | 1.04 | 1.28 | 5.36E-02 |
| All | All_ADHD_Eur_1.00 | BPSA | C | 0.12 | 1.13 | 1.03 | 1.24 | 6.73E-02 |
| All | All_Worry_Subcluster_1.00 | BPNSA | C | 0.11 | 1.12 | 1.02 | 1.22 | 9.53E-02 |
| All | All_PTSD_Male_Derived_1.00 | BPS | BPNSA | 0.20 | 1.22 | 1.03 | 1.44 | 9.65E-02 |
| All | All_Worry_Subcluster_1.00 | BPS | C | 0.14 | 1.15 | 1.02 | 1.31 | 1.14E-01 |
| All | All_PTSD_Male_Derived_1.00 | BPS | BPSA | 0.20 | 1.23 | 1.03 | 1.47 | 1.10E-01 |
| All | All_Insomnia_Daytime_Napping_1.00 | BPS | BPSA | 0.16 | 1.17 | 1.02 | 1.34 | 1.12E-01 |
| All | All_Suicide_Attempt_in_BP_no_NIMH_1.00 | BPS | BPSA | -0.16 | 0.85 | 0.74 | 0.98 | 1.10E-01 |
| All | All_PTSD_1.00 | BPSA | C | 0.12 | 1.13 | 1.01 | 1.27 | 1.11E-01 |
| All | All_Insomnia_Sleep_Duration_1.00 | BPSA | BPNSA | -0.11 | 0.89 | 0.81 | 0.99 | 1.08E-01 |
| All | All_PTSD_1.00 | BPS | BPNSA | 0.18 | 1.19 | 1.02 | 1.40 | 1.17E-01 |
| All | All_Anxiety_1.00 | BPS | C | 0.12 | 1.13 | 1.01 | 1.26 | 1.13E-01 |
| All | All_ADHD_Eur_1.00 | BPSA | BPNSA | 0.11 | 1.12 | 1.01 | 1.24 | 1.27E-01 |
| SexGrp | PRS_Set | Group1 | Group2 | Beta | OR | OR95L | OR95U | Corrected_P |
| All | All_Insomnia_Daytime_Napping_1.00 | BPS | C | 0.13 | 1.14 | 1.00 | 1.29 | 1.63E-01 |
| All | All_Insomnia_1.00 | BPSA | C | 0.10 | 1.10 | 1.00 | 1.21 | 1.58E-01 |
| All | All_ADHD_Male_Derived_1.00 | BPS | BPSA | -0.14 | 0.87 | 0.76 | 1.00 | 1.67E-01 |
| All | All_Insomnia_1.00 | BPS | C | 0.12 | 1.13 | 0.99 | 1.28 | 1.96E-01 |
| All | All_PTSD_Female_Derived_1.00 | BPS | BPNSA | 0.14 | 1.15 | 0.99 | 1.32 | 1.93E-01 |
| All | All_Insomnia_Ease_of_Getting_Up_1.00 | BPNSA | C | -0.09 | 0.92 | 0.84 | 1.01 | 1.94E-01 |
| All | All_ADHD_Female_Derived_1.00 | BPS | BPNSA | 0.13 | 1.14 | 0.99 | 1.31 | 2.00E-01 |
| All | All_ADHD_All_1.00 | BPSA | BPNSA | 0.09 | 1.10 | 0.99 | 1.21 | 2.10E-01 |
| All | All_Insomnia_Snoring_1.00 | BPSA | BPNSA | -0.09 | 0.91 | 0.82 | 1.01 | 2.29E-01 |
| All | All_Insomnia_Daytime_Dozing_1.00 | BPSA | BPNSA | -0.08 | 0.92 | 0.83 | 1.01 | 2.58E-01 |
| All | All_Insomnia_Daytime_Dozing_1.00 | BPSA | C | -0.08 | 0.93 | 0.84 | 1.02 | 2.87E-01 |
| All | All_ADHD_Eur_1.00 | BPS | BPSA | -0.11 | 0.89 | 0.78 | 1.03 | 3.02E-01 |
| All | All_ADHD_Female_Derived_1.00 | BPSA | C | 0.08 | 1.08 | 0.98 | 1.20 | 3.03E-01 |
| All | All_ADHD_Male_Derived_1.00 | BPSA | C | 0.07 | 1.08 | 0.98 | 1.18 | 2.99E-01 |
| All | All_Insomnia_Morningness_1.00 | BPSA | C | -0.07 | 0.93 | 0.84 | 1.02 | 3.01E-01 |
| All | All_PTSD_1.00 | BPSA | BPNSA | 0.09 | 1.10 | 0.97 | 1.24 | 2.99E-01 |
| All | All_Insomnia_Sleep_Duration_1.00 | BPS | BPSA | 0.10 | 1.11 | 0.97 | 1.27 | 3.02E-01 |
| All | All_Insomnia_1.00 | BPSA | BPNSA | 0.07 | 1.08 | 0.97 | 1.19 | 3.33E-01 |
| All | All_ADHD_Male_Derived_1.00 | BPS | BPNSA | -0.09 | 0.91 | 0.80 | 1.05 | 4.20E-01 |
| All | All_PTSD_Female_Derived_1.00 | BPNSA | C | 0.07 | 1.07 | 0.97 | 1.18 | 4.16E-01 |
| All | All_ADHD_All_1.00 | BPS | C | 0.08 | 1.09 | 0.96 | 1.24 | 4.19E-01 |
| All | All_Suicide_attempt_in_MDD_1.00 | BPNSA | C | -0.08 | 0.92 | 0.82 | 1.04 | 4.16E-01 |
| All | All_ADHD_Male_Derived_1.00 | BPSA | BPNSA | 0.06 | 1.07 | 0.96 | 1.18 | 4.54E-01 |
| All | All_ADHD_Female_Derived_1.00 | BPS | BPSA | 0.09 | 1.09 | 0.94 | 1.27 | 4.80E-01 |
| All | All_Suicide_Attempt_in_BP_no_NIMH_1.00 | BPS | C | 0.09 | 1.09 | 0.94 | 1.27 | 4.97E-01 |
| All | All_Insomnia_Daytime_Dozing_1.00 | BPS | BPNSA | -0.08 | 0.93 | 0.81 | 1.06 | 4.91E-01 |
| All | All_ADHD_All_1.00 | BPNSA | C | 0.05 | 1.06 | 0.96 | 1.16 | 4.83E-01 |
| All | All_ADHD_Female_Derived_1.00 | BPSA | BPNSA | 0.06 | 1.06 | 0.96 | 1.19 | 4.81E-01 |
| All | All_Insomnia_Sleep_Duration_1.00 | BPSA | C | -0.05 | 0.95 | 0.86 | 1.04 | 4.75E-01 |
| All | All_ADHD_All_1.00 | BPS | BPSA | -0.08 | 0.92 | 0.80 | 1.06 | 4.73E-01 |
| All | All_Insomnia_Snoring_1.00 | BPS | BPSA | 0.07 | 1.07 | 0.94 | 1.23 | 5.59E-01 |
| All | All_Insomnia_Snoring_1.00 | BPSA | C | -0.05 | 0.95 | 0.87 | 1.05 | 5.91E-01 |
| All | All_Insomnia_1.00 | BPNSA | C | 0.04 | 1.05 | 0.95 | 1.15 | 6.05E-01 |
| SexGrp | PRS_Set | Group1 | Group2 | Beta | OR | OR95L | OR95U | Corrected_P |
| All | All_Insomnia_Daytime_Napping_1.00 | BPS | BPNSA | 0.07 | 1.07 | 0.93 | 1.22 | 5.98E-01 |
| All | All_Suicide_Death_1.00 | BPSA | BPNSA | 0.05 | 1.05 | 0.95 | 1.16 | 6.45E-01 |
| All | All_Insomnia_Ease_of_Getting_Up_1.00 | BPSA | C | -0.04 | 0.96 | 0.87 | 1.05 | 6.45E-01 |
| All | All_Insomnia_Daytime_Napping_1.00 | BPNSA | C | 0.04 | 1.04 | 0.95 | 1.14 | 6.40E-01 |
| All | All_Insomnia_1.00 | BPS | BPNSA | 0.06 | 1.06 | 0.93 | 1.20 | 6.45E-01 |
| All | All_Suicide_attempt_in_MDD_1.00 | BPSA | C | -0.05 | 0.95 | 0.84 | 1.07 | 6.40E-01 |
| All | All_Insomnia_Snoring_1.00 | BPNSA | C | 0.04 | 1.04 | 0.95 | 1.14 | 6.46E-01 |
| All | All_Insomnia_Morningness_1.00 | BPNSA | C | -0.04 | 0.96 | 0.88 | 1.06 | 6.38E-01 |
| All | All_Insomnia_Sleep_Duration_1.00 | BPNSA | C | 0.04 | 1.04 | 0.95 | 1.14 | 6.39E-01 |
| All | All_ADHD_Eur_1.00 | BPS | C | 0.05 | 1.05 | 0.93 | 1.20 | 6.33E-01 |
| All | All_Insomnia_Ease_of_Getting_Up_1.00 | BPSA | BPNSA | 0.04 | 1.04 | 0.94 | 1.16 | 6.32E-01 |
| All | All_PTSD_Female_Derived_1.00 | BPS | BPSA | -0.06 | 0.94 | 0.81 | 1.09 | 6.28E-01 |
| All | All_ADHD_Eur_1.00 | BPNSA | C | 0.04 | 1.04 | 0.95 | 1.14 | 6.21E-01 |
| All | All_Insomnia_Daytime_Napping_1.00 | BPSA | BPNSA | -0.04 | 0.96 | 0.87 | 1.06 | 6.38E-01 |
| All | All_Insomnia_Ease_of_Getting_Up_1.00 | BPS | C | -0.05 | 0.95 | 0.84 | 1.08 | 6.59E-01 |
| All | All_PTSD_1.00 | BPNSA | C | 0.04 | 1.04 | 0.94 | 1.16 | 6.57E-01 |
| All | All_Suicide_Death_1.00 | BPSA | C | 0.03 | 1.04 | 0.94 | 1.14 | 6.69E-01 |
| All | All_Worry_Subcluster_1.00 | BPSA | BPNSA | 0.04 | 1.04 | 0.94 | 1.15 | 6.65E-01 |
| All | All_Insomnia_Daytime_Napping_1.00 | BPSA | C | -0.03 | 0.97 | 0.88 | 1.06 | 6.57E-01 |
| All | All_ADHD_Male_Derived_1.00 | BPNSA | C | 0.03 | 1.03 | 0.94 | 1.13 | 6.70E-01 |
| All | All_PTSD_Male_Derived_1.00 | BPSA | C | 0.04 | 1.04 | 0.93 | 1.16 | 6.82E-01 |
| All | All_PTSD_1.00 | BPS | BPSA | 0.05 | 1.06 | 0.89 | 1.25 | 6.79E-01 |
| All | All_Anxiety_1.00 | BPS | BPNSA | -0.05 | 0.95 | 0.81 | 1.12 | 6.93E-01 |
| All | All_Insomnia_Morningness_1.00 | BPS | BPNSA | 0.04 | 1.04 | 0.92 | 1.18 | 6.88E-01 |
| All | All_ADHD_Male_Derived_1.00 | BPS | C | -0.04 | 0.96 | 0.85 | 1.09 | 6.93E-01 |
| All | All_Anxiety_1.00 | BPS | BPSA | -0.05 | 0.95 | 0.80 | 1.13 | 7.05E-01 |
| All | All_Worry_Subcluster_1.00 | BPS | BPSA | -0.04 | 0.96 | 0.84 | 1.11 | 7.29E-01 |
| All | All_ADHD_Female_Derived_1.00 | BPNSA | C | 0.03 | 1.03 | 0.93 | 1.13 | 7.27E-01 |
| All | All_Insomnia_Daytime_Dozing_1.00 | BPNSA | C | 0.02 | 1.03 | 0.94 | 1.12 | 7.23E-01 |
| All | All_Insomnia_Daytime_Dozing_1.00 | BPS | C | -0.03 | 0.97 | 0.85 | 1.10 | 7.38E-01 |
| All | All_Insomnia_Morningness_1.00 | BPS | BPSA | 0.03 | 1.03 | 0.91 | 1.18 | 7.43E-01 |
| All | All_Suicide_attempt_in_MDD_1.00 | BPSA | BPNSA | 0.03 | 1.03 | 0.91 | 1.18 | 7.55E-01 |
| All | All_Insomnia_Morningness_1.00 | BPSA | BPNSA | -0.02 | 0.98 | 0.89 | 1.08 | 7.49E-01 |
| SexGrp | PRS_Set | Group1 | Group2 | Beta | OR | OR95L | OR95U | Corrected_P |
| All | All_Insomnia_Ease_of_Getting_Up_1.00 | BPS | BPNSA | 0.03 | 1.03 | 0.90 | 1.18 | 7.45E-01 |
| All | All_PTSD_Male_Derived_1.00 | BPNSA | C | 0.02 | 1.02 | 0.92 | 1.14 | 7.49E-01 |
| All | All_ADHD_Eur_1.00 | BPS | BPNSA | -0.03 | 0.97 | 0.85 | 1.11 | 7.49E-01 |
| All | All_PTSD_Male_Derived_1.00 | BPSA | BPNSA | 0.03 | 1.03 | 0.91 | 1.16 | 7.68E-01 |
| All | All_Suicide_Death_1.00 | BPNSA | C | -0.02 | 0.98 | 0.90 | 1.08 | 8.11E-01 |
| All | All_Insomnia_Snoring_1.00 | BPS | BPNSA | -0.02 | 0.98 | 0.85 | 1.12 | 8.09E-01 |
| All | All_Insomnia_Sleep_Duration_1.00 | BPS | BPNSA | -0.02 | 0.98 | 0.86 | 1.12 | 8.19E-01 |
| All | All_Insomnia_Sleep_Duration_1.00 | BPS | C | 0.02 | 1.02 | 0.89 | 1.16 | 8.63E-01 |
| All | All_Worry_Subcluster_1.00 | BPS | BPNSA | 0.02 | 1.02 | 0.89 | 1.16 | 8.69E-01 |
| All | All_ADHD_All_1.00 | BPS | BPNSA | -0.02 | 0.98 | 0.86 | 1.12 | 8.62E-01 |
| All | All_Insomnia_Snoring_1.00 | BPS | C | 0.01 | 1.01 | 0.89 | 1.15 | 8.83E-01 |
| All | All_Anxiety_1.00 | BPSA | BPNSA | 0.01 | 1.01 | 0.89 | 1.15 | 8.76E-01 |
| All | All_Insomnia_Morningness_1.00 | BPS | C | 0.01 | 1.01 | 0.89 | 1.15 | 8.82E-01 |
| All | All_Insomnia_1.00 | BPS | BPSA | -0.01 | 0.99 | 0.86 | 1.13 | 8.87E-01 |
| All | All_Insomnia_Daytime_Dozing_1.00 | BPS | BPSA | 0.01 | 1.01 | 0.88 | 1.16 | 8.93E-01 |
| All | All_Insomnia_Ease_of_Getting_Up_1.00 | BPS | BPSA | -0.01 | 0.99 | 0.87 | 1.14 | 9.16E-01 |
| Group key | |  |  |  |  |  |  |  |
| BPS | individuals with bipolar disorder who died by suicide | | | | | | | |
| BPSA | individuals with bipolar disorder who have a history of one or more suicide attempts | | | | | | | |
| BPNSA | individuals with bipolar disorder who have no history of a suicide attempt | | | | | | | |
| C | Comparison group of Individuals without common psychiatric diagnoses based on self-report ^15^ | | | | | | | |
| Note: Significant results after corrections are highlighted in blue | | | | | | | | |

| Table S7: Sex-Specific PRS Analysis Results | | | | | | | | |
| --- | --- | --- | --- | --- | --- | --- | --- | --- |
| SexGrp | PRS_Set | Group1 | Group2 | Beta | OR | OR95L | OR95U | Corrected_P |
| Male | Male_Suicide_Attempt_in_BP_no_NIMH_1.00 | BPSA | BPNSA | 0.53 | 1.70 | 1.43 | 2.01 | 1.78E-07 |
| Male | Male_Suicide_Death_1.00 | BPS | BPNSA | 0.54 | 1.72 | 1.43 | 2.07 | 6.81E-07 |
| Male | Male_Suicide_Death_1.00 | BPS | C | 0.50 | 1.66 | 1.39 | 1.97 | 8.50E-07 |
| Female | Female_Suicide_Death_1.00 | BPS | BPNSA | 0.51 | 1.67 | 1.37 | 2.05 | 3.42E-05 |
| Female | Female_Suicide_Death_1.00 | BPS | BPSA | 0.52 | 1.68 | 1.37 | 2.06 | 2.98E-05 |
| Female | Female_Suicide_Death_1.00 | BPS | C | 0.50 | 1.65 | 1.35 | 2.01 | 2.77E-05 |
| Male | Male_Suicide_Death_1.00 | BPS | BPSA | 0.47 | 1.60 | 1.31 | 1.96 | 1.49E-04 |
| Male | Male_Suicide_Attempt_in_BP_no_NIMH_1.00 | BPNSA | C | -0.34 | 0.71 | 0.62 | 0.83 | 1.39E-04 |
| Male | Male_Suicide_Attempt_in_BP_no_NIMH_1.00 | BPS | BPNSA | 0.47 | 1.59 | 1.30 | 1.95 | 1.58E-04 |
| Female | Female_Suicide_Attempt_in_BP_no_NIMH_1.00 | BPSA | BPNSA | 0.29 | 1.34 | 1.18 | 1.53 | 2.45E-04 |
| Male | Male_Suicide_attempt_in_MDD_1.00 | BPS | C | 0.46 | 1.59 | 1.25 | 2.01 | 2.28E-03 |
| Female | Female_Suicide_Attempt_in_BP_no_NIMH_1.00 | BPNSA | C | -0.28 | 0.76 | 0.65 | 0.87 | 2.31E-03 |
| Male | Male_Suicide_attempt_in_MDD_1.00 | BPS | BPNSA | 0.42 | 1.51 | 1.19 | 1.93 | 1.26E-02 |
| Male | Male_Worry_Subcluster_1.00 | BPSA | C | 0.26 | 1.29 | 1.11 | 1.51 | 1.87E-02 |
| Male | Male_Suicide_Attempt_in_BP_no_NIMH_1.00 | BPSA | C | 0.25 | 1.28 | 1.10 | 1.49 | 1.93E-02 |
| Female | Female_Anxiety_1.00 | BPSA | C | 0.18 | 1.19 | 1.06 | 1.34 | 3.17E-02 |
| Male | Male_Suicide_Attempt_in_BP_no_NIMH_1.00 | BPS | BPSA | -0.31 | 0.74 | 0.60 | 0.90 | 4.16E-02 |
| Female | Female_Suicide_Attempt_in_BP_no_NIMH_1.00 | BPS | BPNSA | 0.34 | 1.40 | 1.11 | 1.76 | 4.30E-02 |
| Male | Male_Anxiety_1.00 | BPNSA | C | 0.19 | 1.20 | 1.06 | 1.37 | 4.33E-02 |
| Male | Male_Insomnia_Daytime_Napping_1.00 | BPS | BPSA | 0.30 | 1.35 | 1.10 | 1.65 | 4.25E-02 |
| Female | Female_PTSD_Female_Derived_1.00 | BPSA | C | 0.16 | 1.18 | 1.04 | 1.34 | 1.08E-01 |
| Male | Male_ADHD_Eur_1.00 | BPSA | C | 0.20 | 1.22 | 1.05 | 1.42 | 1.07E-01 |
| Male | Male_Suicide_attempt_in_MDD_1.00 | BPS | BPSA | 0.33 | 1.39 | 1.08 | 1.80 | 1.03E-01 |
| Female | Female_PTSD_1.00 | BPS | C | 0.30 | 1.35 | 1.07 | 1.70 | 9.95E-02 |
| Male | Male_Insomnia_Daytime_Napping_1.00 | BPNSA | C | 0.17 | 1.18 | 1.03 | 1.35 | 1.24E-01 |
| Male | Male_Insomnia_Daytime_Napping_1.00 | BPSA | BPNSA | -0.20 | 0.82 | 0.69 | 0.96 | 1.26E-01 |
| Male | Male_Insomnia_Daytime_Napping_1.00 | BPS | C | 0.20 | 1.23 | 1.04 | 1.45 | 1.38E-01 |
| Male | Male_ADHD_All_1.00 | BPSA | C | 0.18 | 1.20 | 1.03 | 1.40 | 1.52E-01 |
| Male | Male_PTSD_Male_Derived_1.00 | BPS | C | 0.24 | 1.27 | 1.03 | 1.57 | 1.70E-01 |
| Female | Female_PTSD_Female_Derived_1.00 | BPSA | BPNSA | 0.16 | 1.17 | 1.02 | 1.34 | 1.71E-01 |
| Male | Male_PTSD_1.00 | BPS | C | 0.23 | 1.25 | 1.02 | 1.54 | 1.92E-01 |
| Male | Male_Insomnia_1.00 | BPSA | C | 0.17 | 1.18 | 1.01 | 1.38 | 2.04E-01 |
| SexGrp | PRS_Set | Group1 | Group2 | Beta | OR | OR95L | OR95U | Corrected_P |
| Female | Female_PTSD_Female_Derived_1.00 | BPS | C | 0.22 | 1.25 | 1.01 | 1.54 | 2.29E-01 |
| Female | Female_Insomnia_Morningness_1.00 | BPSA | C | -0.13 | 0.88 | 0.78 | 0.99 | 2.43E-01 |
| Female | Female_PTSD_Female_Derived_1.00 | BPS | BPNSA | 0.22 | 1.25 | 1.00 | 1.56 | 2.80E-01 |
| Male | Male_Worry_Subcluster_1.00 | BPS | C | 0.17 | 1.18 | 1.00 | 1.39 | 2.74E-01 |
| Female | Female_Insomnia_Daytime_Dozing_1.00 | BPSA | C | -0.12 | 0.89 | 0.79 | 1.00 | 2.91E-01 |
| Female | Female_Suicide_Attempt_in_BP_no_NIMH_1.00 | BPSA | C | 0.12 | 1.13 | 1.00 | 1.28 | 3.20E-01 |
| Female | Female_ADHD_Eur_1.00 | BPSA | BPNSA | 0.12 | 1.13 | 0.99 | 1.29 | 3.55E-01 |
| Male | Male_ADHD_Male_Derived_1.00 | BPSA | C | 0.14 | 1.15 | 0.99 | 1.34 | 3.47E-01 |
| Male | Male_Anxiety_1.00 | BPSA | BPNSA | -0.19 | 0.83 | 0.68 | 1.02 | 3.56E-01 |
| Female | Female_PTSD_1.00 | BPS | BPNSA | 0.22 | 1.25 | 0.98 | 1.59 | 3.60E-01 |
| Male | Male_Insomnia_1.00 | BPS | C | 0.15 | 1.16 | 0.98 | 1.37 | 3.77E-01 |
| Male | Male_Worry_Subcluster_1.00 | BPNSA | C | 0.12 | 1.12 | 0.99 | 1.28 | 3.75E-01 |
| Female | Female_Worry_Subcluster_1.00 | BPSA | C | 0.11 | 1.12 | 0.99 | 1.26 | 3.67E-01 |
| Female | Female_ADHD_Female_Derived_1.00 | BPS | C | 0.18 | 1.20 | 0.98 | 1.47 | 3.69E-01 |
| Male | Male_ADHD_All_1.00 | BPS | C | 0.14 | 1.16 | 0.97 | 1.37 | 4.36E-01 |
| Female | Female_Anxiety_1.00 | BPSA | BPNSA | 0.13 | 1.14 | 0.97 | 1.34 | 4.29E-01 |
| Male | Male_Insomnia_Snoring_1.00 | BPS | BPSA | 0.17 | 1.18 | 0.97 | 1.45 | 4.28E-01 |
| Female | Female_PTSD_1.00 | BPSA | C | 0.12 | 1.12 | 0.98 | 1.29 | 4.20E-01 |
| Male | Male_Insomnia_Daytime_Dozing_1.00 | BPS | BPNSA | -0.14 | 0.87 | 0.73 | 1.03 | 4.13E-01 |
| Male | Male_Insomnia_Sleep_Duration_1.00 | BPSA | BPNSA | -0.13 | 0.88 | 0.75 | 1.03 | 4.12E-01 |
| Female | Female_ADHD_Eur_1.00 | BPS | BPSA | -0.16 | 0.85 | 0.69 | 1.04 | 4.08E-01 |
| Female | Female_Suicide_attempt_in_MDD_1.00 | BPS | BPNSA | 0.23 | 1.26 | 0.95 | 1.67 | 4.08E-01 |
| Female | Female_Worry_Subcluster_1.00 | BPNSA | C | 0.10 | 1.11 | 0.97 | 1.26 | 4.36E-01 |
| Female | Female_ADHD_All_1.00 | BPSA | BPNSA | 0.10 | 1.11 | 0.97 | 1.26 | 4.37E-01 |
| Male | Male_PTSD_1.00 | BPSA | C | 0.14 | 1.15 | 0.96 | 1.38 | 4.40E-01 |
| Female | Female_Suicide_attempt_in_MDD_1.00 | BPS | BPSA | 0.20 | 1.22 | 0.94 | 1.58 | 4.71E-01 |
| Female | Female_Anxiety_1.00 | BPNSA | C | 0.09 | 1.09 | 0.97 | 1.23 | 4.70E-01 |
| Female | Female_Anxiety_1.00 | BPS | C | 0.13 | 1.13 | 0.96 | 1.34 | 4.65E-01 |
| Female | Female_Insomnia_Sleep_Duration_1.00 | BPSA | BPNSA | -0.10 | 0.91 | 0.80 | 1.03 | 4.58E-01 |
| Male | Male_Insomnia_Ease_of_Getting_Up_1.00 | BPS | C | -0.13 | 0.88 | 0.74 | 1.04 | 4.53E-01 |
| Male | Male_Anxiety_1.00 | BPS | C | 0.12 | 1.12 | 0.96 | 1.31 | 4.47E-01 |
| Male | Male_Insomnia_1.00 | BPSA | BPNSA | 0.12 | 1.13 | 0.96 | 1.32 | 4.69E-01 |
| Female | Female_Insomnia_Daytime_Napping_1.00 | BPS | BPNSA | 0.16 | 1.17 | 0.94 | 1.45 | 4.75E-01 |
| SexGrp | PRS_Set | Group1 | Group2 | Beta | OR | OR95L | OR95U | Corrected_P |
| Male | Male_PTSD_1.00 | BPS | BPNSA | 0.16 | 1.17 | 0.94 | 1.45 | 4.71E-01 |
| Male | Male_ADHD_Eur_1.00 | BPNSA | C | 0.10 | 1.10 | 0.96 | 1.26 | 4.67E-01 |
| Male | Male_Worry_Subcluster_1.00 | BPSA | BPNSA | 0.12 | 1.13 | 0.96 | 1.33 | 4.62E-01 |
| Female | Female_Worry_Subcluster_1.00 | BPS | C | 0.14 | 1.15 | 0.95 | 1.40 | 4.72E-01 |
| Male | Male_Insomnia_Sleep_Duration_1.00 | BPSA | C | -0.11 | 0.90 | 0.77 | 1.04 | 4.72E-01 |
| Male | Male_Insomnia_Ease_of_Getting_Up_1.00 | BPNSA | C | -0.09 | 0.91 | 0.80 | 1.04 | 4.74E-01 |
| Female | Female_Suicide_attempt_in_MDD_1.00 | BPNSA | C | -0.12 | 0.89 | 0.76 | 1.05 | 4.74E-01 |
| Male | Male_ADHD_Eur_1.00 | BPS | C | 0.12 | 1.13 | 0.95 | 1.34 | 4.68E-01 |
| Male | Male_Insomnia_Ease_of_Getting_Up_1.00 | BPSA | C | -0.11 | 0.90 | 0.77 | 1.05 | 4.66E-01 |
| Female | Female_ADHD_All_1.00 | BPSA | C | 0.08 | 1.09 | 0.96 | 1.22 | 4.67E-01 |
| Female | Female_ADHD_All_1.00 | BPS | BPSA | -0.14 | 0.87 | 0.72 | 1.07 | 4.85E-01 |
| Male | Male_Insomnia_Daytime_Dozing_1.00 | BPNSA | C | 0.09 | 1.10 | 0.96 | 1.25 | 4.87E-01 |
| Male | Male_ADHD_All_1.00 | BPNSA | C | 0.09 | 1.09 | 0.96 | 1.25 | 4.91E-01 |
| Female | Female_Insomnia_Daytime_Napping_1.00 | BPNSA | C | -0.09 | 0.92 | 0.81 | 1.04 | 5.04E-01 |
| Male | Male_Insomnia_Snoring_1.00 | BPSA | BPNSA | -0.11 | 0.90 | 0.76 | 1.06 | 5.15E-01 |
| Male | Male_Insomnia_Sleep_Duration_1.00 | BPS | BPSA | 0.12 | 1.13 | 0.94 | 1.36 | 5.14E-01 |
| Female | Female_ADHD_Female_Derived_1.00 | BPS | BPNSA | 0.14 | 1.15 | 0.92 | 1.44 | 5.21E-01 |
| Female | Female_Insomnia_Ease_of_Getting_Up_1.00 | BPNSA | C | -0.08 | 0.92 | 0.81 | 1.05 | 5.29E-01 |
| Female | Female_Insomnia_Snoring_1.00 | BPSA | BPNSA | -0.08 | 0.92 | 0.80 | 1.05 | 5.32E-01 |
| Male | Male_PTSD_Male_Derived_1.00 | BPS | BPNSA | 0.14 | 1.15 | 0.92 | 1.44 | 5.29E-01 |
| Female | Female_Suicide_attempt_in_MDD_1.00 | BPSA | C | -0.09 | 0.91 | 0.78 | 1.06 | 5.27E-01 |
| Male | Male_Insomnia_Daytime_Dozing_1.00 | BPSA | BPNSA | -0.10 | 0.91 | 0.78 | 1.06 | 5.42E-01 |
| Female | Female_PTSD_1.00 | BPSA | BPNSA | 0.09 | 1.09 | 0.94 | 1.27 | 5.59E-01 |
| Male | Male_Anxiety_1.00 | BPSA | C | 0.08 | 1.09 | 0.94 | 1.25 | 5.55E-01 |
| Female | Female_Insomnia_Daytime_Dozing_1.00 | BPSA | BPNSA | -0.08 | 0.93 | 0.81 | 1.05 | 5.53E-01 |
| Male | Male_Anxiety_1.00 | BPS | BPNSA | -0.13 | 0.88 | 0.71 | 1.10 | 5.65E-01 |
| Male | Male_Insomnia_Snoring_1.00 | BPS | C | 0.10 | 1.10 | 0.93 | 1.30 | 5.66E-01 |
| Male | Male_ADHD_Eur_1.00 | BPSA | BPNSA | 0.09 | 1.10 | 0.93 | 1.30 | 5.68E-01 |
| Male | Male_PTSD_Male_Derived_1.00 | BPS | BPSA | 0.14 | 1.15 | 0.90 | 1.48 | 5.72E-01 |
| Male | Male_Suicide_Attempt_in_BP_no_NIMH_1.00 | BPS | C | 0.11 | 1.12 | 0.92 | 1.36 | 5.93E-01 |
| Male | Male_Insomnia_Snoring_1.00 | BPNSA | C | 0.07 | 1.08 | 0.94 | 1.23 | 5.91E-01 |
| Female | Female_Insomnia_Ease_of_Getting_Up_1.00 | BPSA | BPNSA | 0.07 | 1.07 | 0.94 | 1.23 | 6.01E-01 |
| Female | Female_ADHD_Female_Derived_1.00 | BPS | BPSA | 0.12 | 1.12 | 0.91 | 1.39 | 6.03E-01 |
| SexGrp | PRS_Set | Group1 | Group2 | Beta | OR | OR95L | OR95U | Corrected_P |
| Female | Female_Insomnia_Morningness_1.00 | BPNSA | C | -0.07 | 0.93 | 0.82 | 1.06 | 6.05E-01 |
| Male | Male_Suicide_Death_1.00 | BPSA | BPNSA | 0.09 | 1.09 | 0.92 | 1.29 | 6.14E-01 |
| Male | Male_Suicide_Death_1.00 | BPSA | C | 0.08 | 1.09 | 0.93 | 1.27 | 6.16E-01 |
| Female | Female_Insomnia_Snoring_1.00 | BPSA | C | -0.06 | 0.94 | 0.83 | 1.06 | 6.25E-01 |
| Female | Female_ADHD_Eur_1.00 | BPSA | C | 0.06 | 1.06 | 0.94 | 1.20 | 6.27E-01 |
| Female | Female_Insomnia_Sleep_Duration_1.00 | BPNSA | C | 0.07 | 1.07 | 0.94 | 1.22 | 6.21E-01 |
| Male | Male_PTSD_1.00 | BPSA | BPNSA | 0.10 | 1.10 | 0.91 | 1.33 | 6.35E-01 |
| Male | Male_ADHD_All_1.00 | BPSA | BPNSA | 0.08 | 1.08 | 0.92 | 1.27 | 6.39E-01 |
| Female | Female_Insomnia_Snoring_1.00 | BPS | BPNSA | -0.10 | 0.90 | 0.73 | 1.12 | 6.71E-01 |
| Male | Male_ADHD_Male_Derived_1.00 | BPNSA | C | 0.06 | 1.07 | 0.93 | 1.22 | 6.69E-01 |
| Female | Female_Insomnia_Snoring_1.00 | BPS | C | -0.09 | 0.91 | 0.74 | 1.11 | 6.65E-01 |
| Female | Female_PTSD_1.00 | BPS | BPSA | 0.11 | 1.12 | 0.88 | 1.42 | 6.60E-01 |
| Female | Female_Insomnia_Sleep_Duration_1.00 | BPS | BPSA | 0.09 | 1.10 | 0.90 | 1.34 | 6.59E-01 |
| Male | Male_ADHD_Male_Derived_1.00 | BPSA | BPNSA | 0.07 | 1.08 | 0.92 | 1.27 | 6.72E-01 |
| Male | Male_Worry_Subcluster_1.00 | BPS | BPSA | -0.09 | 0.91 | 0.75 | 1.12 | 6.80E-01 |
| Female | Female_Insomnia_Daytime_Napping_1.00 | BPSA | BPNSA | 0.06 | 1.06 | 0.93 | 1.20 | 6.76E-01 |
| Female | Female_Suicide_attempt_in_MDD_1.00 | BPS | C | 0.11 | 1.12 | 0.87 | 1.44 | 6.78E-01 |
| Male | Male_ADHD_Male_Derived_1.00 | BPS | BPSA | -0.09 | 0.92 | 0.76 | 1.12 | 6.84E-01 |
| Female | Female_Insomnia_1.00 | BPSA | C | 0.05 | 1.05 | 0.93 | 1.19 | 7.15E-01 |
| Female | Female_Anxiety_1.00 | BPS | BPSA | -0.10 | 0.90 | 0.71 | 1.15 | 7.19E-01 |
| Female | Female_Insomnia_1.00 | BPS | C | 0.08 | 1.08 | 0.89 | 1.31 | 7.29E-01 |
| Female | Female_Insomnia_Sleep_Duration_1.00 | BPS | C | 0.08 | 1.08 | 0.89 | 1.32 | 7.40E-01 |
| Female | Female_Insomnia_1.00 | BPS | BPNSA | 0.08 | 1.08 | 0.88 | 1.33 | 7.45E-01 |
| Female | Female_Insomnia_1.00 | BPNSA | C | 0.05 | 1.05 | 0.92 | 1.19 | 7.76E-01 |
| Male | Male_ADHD_Male_Derived_1.00 | BPS | BPNSA | -0.07 | 0.94 | 0.78 | 1.12 | 7.90E-01 |
| Male | Male_Insomnia_Morningness_1.00 | BPS | BPNSA | 0.06 | 1.06 | 0.90 | 1.26 | 7.85E-01 |
| Male | Male_Insomnia_Daytime_Napping_1.00 | BPSA | C | -0.05 | 0.95 | 0.81 | 1.10 | 7.84E-01 |
| Female | Female_Insomnia_Ease_of_Getting_Up_1.00 | BPS | BPNSA | 0.07 | 1.07 | 0.88 | 1.32 | 8.03E-01 |
| Female | Female_Insomnia_Morningness_1.00 | BPSA | BPNSA | -0.04 | 0.96 | 0.84 | 1.09 | 7.97E-01 |
| Female | Female_PTSD_1.00 | BPNSA | C | 0.05 | 1.05 | 0.91 | 1.22 | 7.97E-01 |
| Female | Female_ADHD_Female_Derived_1.00 | BPSA | C | 0.04 | 1.04 | 0.92 | 1.19 | 8.14E-01 |
| Male | Male_ADHD_Eur_1.00 | BPS | BPSA | -0.07 | 0.94 | 0.77 | 1.14 | 8.12E-01 |
| Male | Male_Insomnia_1.00 | BPS | BPNSA | 0.05 | 1.06 | 0.89 | 1.25 | 8.20E-01 |
| SexGrp | PRS_Set | Group1 | Group2 | Beta | OR | OR95L | OR95U | Corrected_P |
| Female | Female_Insomnia_Morningness_1.00 | BPS | BPSA | 0.06 | 1.06 | 0.88 | 1.28 | 8.15E-01 |
| Female | Female_Insomnia_1.00 | BPSA | BPNSA | 0.04 | 1.04 | 0.92 | 1.19 | 8.10E-01 |
| Male | Male_PTSD_Male_Derived_1.00 | BPSA | C | 0.06 | 1.06 | 0.88 | 1.27 | 8.05E-01 |
| Female | Female_Insomnia_Daytime_Napping_1.00 | BPS | BPSA | 0.06 | 1.06 | 0.87 | 1.30 | 8.11E-01 |
| Female | Female_Insomnia_Daytime_Dozing_1.00 | BPS | BPSA | 0.06 | 1.06 | 0.87 | 1.30 | 8.14E-01 |
| Female | Female_Insomnia_Daytime_Dozing_1.00 | BPNSA | C | -0.04 | 0.96 | 0.85 | 1.09 | 8.19E-01 |
| Male | Male_Suicide_attempt_in_MDD_1.00 | BPSA | BPNSA | 0.06 | 1.06 | 0.87 | 1.31 | 8.20E-01 |
| Male | Male_Insomnia_Daytime_Dozing_1.00 | BPS | C | -0.05 | 0.95 | 0.80 | 1.13 | 8.24E-01 |
| Female | Female_Insomnia_Ease_of_Getting_Up_1.00 | BPS | C | 0.06 | 1.06 | 0.87 | 1.28 | 8.37E-01 |
| Male | Male_ADHD_Male_Derived_1.00 | BPS | C | 0.05 | 1.05 | 0.89 | 1.24 | 8.38E-01 |
| Male | Male_Insomnia_Ease_of_Getting_Up_1.00 | BPS | BPSA | -0.05 | 0.95 | 0.78 | 1.15 | 8.54E-01 |
| Female | Female_ADHD_Female_Derived_1.00 | BPSA | BPNSA | 0.04 | 1.04 | 0.90 | 1.20 | 8.65E-01 |
| Female | Female_Suicide_Attempt_in_BP_no_NIMH_1.00 | BPS | C | 0.06 | 1.06 | 0.84 | 1.34 | 8.83E-01 |
| Male | Male_Insomnia_Sleep_Duration_1.00 | BPS | BPNSA | -0.04 | 0.96 | 0.80 | 1.14 | 8.97E-01 |
| Female | Female_Insomnia_Daytime_Napping_1.00 | BPS | C | 0.05 | 1.05 | 0.86 | 1.28 | 8.95E-01 |
| Female | Female_ADHD_Eur_1.00 | BPNSA | C | -0.03 | 0.97 | 0.85 | 1.10 | 8.93E-01 |
| Female | Female_Insomnia_Ease_of_Getting_Up_1.00 | BPS | BPSA | 0.05 | 1.05 | 0.86 | 1.27 | 8.92E-01 |
| Female | Female_ADHD_Eur_1.00 | BPS | C | -0.05 | 0.96 | 0.79 | 1.16 | 8.86E-01 |
| Female | Female_Insomnia_Daytime_Napping_1.00 | BPSA | C | -0.03 | 0.97 | 0.86 | 1.10 | 8.84E-01 |
| Male | Male_Insomnia_1.00 | BPNSA | C | 0.03 | 1.03 | 0.90 | 1.18 | 8.79E-01 |
| Male | Male_Suicide_attempt_in_MDD_1.00 | BPNSA | C | -0.04 | 0.96 | 0.81 | 1.15 | 8.83E-01 |
| Male | Male_Insomnia_Morningness_1.00 | BPS | C | 0.04 | 1.04 | 0.88 | 1.23 | 8.95E-01 |
| Female | Female_Anxiety_1.00 | BPS | BPNSA | 0.05 | 1.05 | 0.82 | 1.35 | 8.97E-01 |
| Male | Male_ADHD_Eur_1.00 | BPS | BPNSA | -0.03 | 0.97 | 0.81 | 1.15 | 9.29E-01 |
| Male | Male_ADHD_All_1.00 | BPS | BPSA | -0.04 | 0.96 | 0.79 | 1.18 | 9.26E-01 |
| Female | Female_Insomnia_Morningness_1.00 | BPS | C | -0.04 | 0.96 | 0.79 | 1.17 | 9.24E-01 |
| Male | Male_Insomnia_Snoring_1.00 | BPS | BPNSA | 0.03 | 1.03 | 0.86 | 1.24 | 9.28E-01 |
| Male | Male_PTSD_1.00 | BPNSA | C | 0.03 | 1.03 | 0.88 | 1.20 | 9.29E-01 |
| Male | Male_Suicide_attempt_in_MDD_1.00 | BPSA | C | 0.03 | 1.04 | 0.85 | 1.26 | 9.28E-01 |
| Female | Female_Insomnia_Daytime_Dozing_1.00 | BPS | BPNSA | 0.04 | 1.04 | 0.84 | 1.29 | 9.29E-01 |
| Female | Female_Worry_Subcluster_1.00 | BPS | BPSA | 0.03 | 1.04 | 0.85 | 1.26 | 9.27E-01 |
| Male | Male_PTSD_Male_Derived_1.00 | BPNSA | C | 0.03 | 1.03 | 0.88 | 1.20 | 9.42E-01 |
| Female | Female_Insomnia_Sleep_Duration_1.00 | BPSA | C | -0.02 | 0.98 | 0.87 | 1.11 | 9.37E-01 |
| SexGrp | PRS_Set | Group1 | Group2 | Beta | OR | OR95L | OR95U | Corrected_P |
| Female | Female_Insomnia_Snoring_1.00 | BPNSA | C | 0.02 | 1.02 | 0.89 | 1.17 | 9.37E-01 |
| Female | Female_Suicide_Death_1.00 | BPSA | BPNSA | 0.02 | 1.02 | 0.90 | 1.16 | 9.39E-01 |
| Male | Male_Worry_Subcluster_1.00 | BPS | BPNSA | 0.03 | 1.03 | 0.86 | 1.22 | 9.42E-01 |
| Female | Female_Suicide_Death_1.00 | BPNSA | C | -0.02 | 0.98 | 0.86 | 1.12 | 9.47E-01 |
| Male | Male_Insomnia_Sleep_Duration_1.00 | BPS | C | -0.02 | 0.98 | 0.82 | 1.16 | 9.52E-01 |
| Male | Male_Insomnia_Morningness_1.00 | BPNSA | C | -0.02 | 0.98 | 0.86 | 1.12 | 9.52E-01 |
| Male | Male_Suicide_Death_1.00 | BPNSA | C | -0.02 | 0.98 | 0.86 | 1.13 | 9.54E-01 |
| Male | Male_Insomnia_Daytime_Dozing_1.00 | BPS | BPSA | -0.02 | 0.98 | 0.81 | 1.18 | 9.48E-01 |
| Female | Female_ADHD_Eur_1.00 | BPS | BPNSA | -0.03 | 0.97 | 0.79 | 1.20 | 9.46E-01 |
| Female | Female_Worry_Subcluster_1.00 | BPSA | BPNSA | -0.02 | 0.98 | 0.86 | 1.12 | 9.50E-01 |
| Female | Female_ADHD_All_1.00 | BPS | BPNSA | -0.02 | 0.98 | 0.79 | 1.20 | 9.54E-01 |
| Female | Female_Worry_Subcluster_1.00 | BPS | BPNSA | 0.02 | 1.02 | 0.84 | 1.25 | 9.57E-01 |
| Male | Male_Insomnia_Daytime_Napping_1.00 | BPS | BPNSA | 0.02 | 1.02 | 0.85 | 1.22 | 9.63E-01 |
| Female | Female_Suicide_Attempt_in_BP_no_NIMH_1.00 | BPS | BPSA | -0.02 | 0.98 | 0.81 | 1.19 | 9.61E-01 |
| Male | Male_PTSD_Male_Derived_1.00 | BPSA | BPNSA | 0.02 | 1.02 | 0.84 | 1.24 | 9.56E-01 |
| Female | Female_Insomnia_Morningness_1.00 | BPS | BPNSA | 0.02 | 1.02 | 0.84 | 1.23 | 9.55E-01 |
| Male | Male_Insomnia_Morningness_1.00 | BPSA | BPNSA | 0.01 | 1.01 | 0.87 | 1.18 | 9.84E-01 |
| Male | Male_Insomnia_Morningness_1.00 | BPSA | C | 0.01 | 1.01 | 0.87 | 1.18 | 9.79E-01 |
| Female | Female_PTSD_Female_Derived_1.00 | BPS | BPSA | 0.02 | 1.02 | 0.83 | 1.25 | 9.78E-01 |
| Female | Female_Suicide_attempt_in_MDD_1.00 | BPSA | BPNSA | 0.01 | 1.01 | 0.85 | 1.20 | 9.80E-01 |
| Male | Male_Insomnia_Morningness_1.00 | BPS | BPSA | 0.01 | 1.01 | 0.84 | 1.22 | 9.75E-01 |
| Female | Female_ADHD_All_1.00 | BPNSA | C | 0.01 | 1.01 | 0.89 | 1.15 | 9.80E-01 |
| Female | Female_PTSD_Female_Derived_1.00 | BPNSA | C | 0.01 | 1.01 | 0.88 | 1.15 | 9.87E-01 |
| Female | Female_Insomnia_Snoring_1.00 | BPS | BPSA | -0.01 | 0.99 | 0.81 | 1.20 | 9.85E-01 |
| Female | Female_ADHD_Female_Derived_1.00 | BPNSA | C | 0.01 | 1.01 | 0.88 | 1.15 | 9.82E-01 |
| Female | Female_Insomnia_Sleep_Duration_1.00 | BPS | BPNSA | 0.01 | 1.01 | 0.82 | 1.25 | 9.77E-01 |
| Male | Male_ADHD_All_1.00 | BPS | BPNSA | -0.01 | 0.99 | 0.83 | 1.18 | 9.78E-01 |
| Male | Male_Insomnia_Snoring_1.00 | BPSA | C | -0.01 | 0.99 | 0.85 | 1.16 | 9.86E-01 |
| Male | Male_Insomnia_Sleep_Duration_1.00 | BPNSA | C | 0.01 | 1.01 | 0.88 | 1.15 | 9.91E-01 |
| Male | Male_Insomnia_Daytime_Dozing_1.00 | BPSA | C | -0.01 | 0.99 | 0.85 | 1.16 | 9.88E-01 |
| Female | Female_Insomnia_Daytime_Dozing_1.00 | BPS | C | -0.01 | 0.99 | 0.82 | 1.21 | 9.98E-01 |
| Male | Male_Anxiety_1.00 | BPS | BPSA | -0.01 | 0.99 | 0.78 | 1.26 | 1.00E+00 |
| Male | Male_Insomnia_Ease_of_Getting_Up_1.00 | BPS | BPNSA | 0.00 | 1.00 | 0.84 | 1.19 | 1.00E+00 |
| SexGrp | PRS_Set | Group1 | Group2 | Beta | OR | OR95L | OR95U | Corrected_P |
| Female | Female_Suicide_Death_1.00 | BPSA | C | 0.00 | 1.00 | 0.88 | 1.13 | 1.00E+00 |
| Male | Male_Insomnia_1.00 | BPS | BPSA | 0.00 | 1.00 | 0.83 | 1.22 | 1.00E+00 |
| Male | Male_Insomnia_Ease_of_Getting_Up_1.00 | BPSA | BPNSA | 0.00 | 1.00 | 0.85 | 1.18 | 1.00E+00 |
| Female | Female_ADHD_All_1.00 | BPS | C | 0.00 | 1.00 | 0.82 | 1.21 | 1.00E+00 |
| Female | Female_Insomnia_1.00 | BPS | BPSA | 0.00 | 1.00 | 0.82 | 1.22 | 1.00E+00 |
| Male | Male_PTSD_1.00 | BPS | BPSA | 0.00 | 1.00 | 0.79 | 1.26 | 1.00E+00 |
| Female | Female_Insomnia_Ease_of_Getting_Up_1.00 | BPSA | C | 0.00 | 1.00 | 0.88 | 1.13 | 9.97E-01 |
| Group key | | | | | | | | |
| BPS | individuals with bipolar disorder who died by suicide | | | | | | | |
| BPSA | individuals with bipolar disorder who have a history of one or more suicide attempts | | | | | | | |
| BPNSA | individuals with bipolar disorder who have no history of a suicide attempt | | | | | | | |
| C | Comparison group of Individuals without several common psychiatric diagnoses based on self-report ^15^ | | | | | | | |
| Note: Significant results after corrections are highlighted in blue | | | | | | | | |

| Table S8: Utah USGRS BPS Versus NBPS PRS Analysis Results | | | | | | |
| --- | --- | --- | --- | --- | --- | --- |
| SexGrp | PRS Set | Beta | OR | OR95L | OR95U | Corrected_P |
| All | All_Insomnia_Daytime_Napping_1.00 | -0.16 | 0.85 | 0.76 | 0.95 | 9.96E-02 |
| All | All_ADHD_Eur_1.00 | 0.13 | 1.14 | 1.02 | 1.28 | 2.13E-01 |
| All | All_ADHD_Male_Derived_1.00 | 0.13 | 1.14 | 1.02 | 1.28 | 1.51E-01 |
| All | All_ADHD_All_1.00 | -0.12 | 0.89 | 0.79 | 1.00 | 2.20E-01 |
| All | All_Insomnia_Snoring_1.00 | -0.11 | 0.90 | 0.80 | 1.01 | 2.37E-01 |
| All | All_Suicide_Attempt_in_BP_1.00 | 0.10 | 1.11 | 0.98 | 1.25 | 2.96E-01 |
| All | All_Worry_Subcluster_1.00 | -0.07 | 0.93 | 0.83 | 1.05 | 6.66E-01 |
| All | All_Insomnia_Daytime_Dozing_1.00 | 0.04 | 1.05 | 0.93 | 1.17 | 1.00E+00 |
| All | All_PTSD_1.00 | 0.04 | 1.04 | 0.92 | 1.18 | 1.00E+00 |
| All | All_Insomnia_Ease_of_Getting_Up_1.00 | 0.04 | 1.04 | 0.93 | 1.16 | 9.64E-01 |
| All | All_Insomnia_Sleep_Duration_1.00 | -0.03 | 0.97 | 0.87 | 1.09 | 1.00E+00 |
| All | All_Anxiety_1.00 | -0.03 | 0.97 | 0.87 | 1.09 | 9.67E-01 |
| All | All_PTSD_Male_Derived_1.00 | 0.03 | 1.03 | 0.91 | 1.16 | 9.82E-01 |
| All | All_Suicide_attempt_in_MDD_1.00 | -0.03 | 0.97 | 0.86 | 1.11 | 9.37E-01 |
| All | All_ADHD_Female_Derived_1.00 | 0.02 | 1.02 | 0.91 | 1.15 | 8.94E-01 |
| All | All_Suicide_Death_1.00 | 0.02 | 1.02 | 0.91 | 1.14 | 8.70E-01 |
| All | All_Insomnia_1.00 | -0.01 | 0.99 | 0.88 | 1.10 | 8.98E-01 |
| All | All_Insomnia_Morningness_1.00 | -0.01 | 0.99 | 0.89 | 1.11 | 9.52E-01 |
| All | All_PTSD_Female_Derived_1.00 | 0.01 | 1.01 | 0.90 | 1.13 | 9.14E-01 |

| Table S9: Utah USGRS BPS Versus NBPS Sex-Specific PRS Analysis Results | | | | | | |
| --- | --- | --- | --- | --- | --- | --- |
| SexGrp | Test | Beta | OR | OR95L | OR95U | Corrected_P |
| Male | Male_Insomnia_Daytime_Napping_1.00 | -0.18 | 0.84 | 0.73 | 0.97 | 4.83E-01 |
| Female | Female_ADHD_Eur_1.00 | 0.24 | 1.27 | 1.04 | 1.56 | 3.37E-01 |
| Male | Male_Insomnia_Snoring_1.00 | -0.15 | 0.86 | 0.75 | 0.99 | 4.16E-01 |
| Female | Female_ADHD_All_1.00 | -0.20 | 0.82 | 0.67 | 1.01 | 4.78E-01 |
| Female | Female_PTSD_Female_Derived_1.00 | -0.17 | 0.85 | 0.69 | 1.04 | 8.00E-01 |
| Female | Female_Insomnia_Daytime_Napping_1.00 | -0.15 | 0.86 | 0.71 | 1.06 | 9.02E-01 |
| Male | Male_Worry_Subcluster_1.00 | -0.09 | 0.91 | 0.79 | 1.05 | 1.00E+00 |
| Male | Male_Suicide_Attempt_in_BP_no_NIMH_1.00 | 0.09 | 1.09 | 0.95 | 1.26 | 9.45E-01 |
| Male | Male_ADHD_Male_Derived_1.00 | 0.08 | 1.08 | 0.94 | 1.24 | 1.00E+00 |
| Female | Female_Suicide_Attempt_in_BP_no_NIMH_1.00 | 0.10 | 1.11 | 0.90 | 1.37 | 1.00E+00 |
| Male | Male_Suicide_attempt_in_MDD_1.00 | -0.08 | 0.93 | 0.79 | 1.09 | 1.00E+00 |
| Male | Male_ADHD_Eur_1.00 | 0.07 | 1.07 | 0.93 | 1.23 | 1.00E+00 |
| Male | Male_PTSD_1.00 | 0.07 | 1.07 | 0.92 | 1.25 | 9.53E-01 |
| Male | Male_Insomnia_Ease_of_Getting_Up_1.00 | 0.06 | 1.06 | 0.92 | 1.22 | 1.00E+00 |
| Male | Male_ADHD_All_1.00 | -0.06 | 0.94 | 0.82 | 1.09 | 9.46E-01 |
| Male | Male_Insomnia_Daytime_Dozing_1.00 | 0.06 | 1.06 | 0.92 | 1.22 | 8.92E-01 |
| Male | Male_Anxiety_1.00 | -0.04 | 0.96 | 0.83 | 1.10 | 1.00E+00 |
| Female | Female_Anxiety_1.00 | 0.06 | 1.06 | 0.87 | 1.30 | 1.00E+00 |
| Female | Female_Suicide_attempt_in_MDD_1.00 | 0.06 | 1.06 | 0.84 | 1.34 | 1.00E+00 |
| Female | Female_Insomnia_Sleep_Duration_1.00 | -0.05 | 0.95 | 0.78 | 1.16 | 1.00E+00 |
| Female | Female_Suicide_Death_1.00 | 0.05 | 1.05 | 0.86 | 1.28 | 1.00E+00 |
| Female | Female_Insomnia_Snoring_1.00 | -0.05 | 0.95 | 0.78 | 1.17 | 1.00E+00 |
| Male | Male_PTSD_Male_Derived_1.00 | 0.03 | 1.03 | 0.88 | 1.21 | 1.00E+00 |
| Female | Female_Insomnia_1.00 | -0.04 | 0.96 | 0.79 | 1.17 | 9.76E-01 |
| Female | Female_ADHD_Female_Derived_1.00 | 0.04 | 1.04 | 0.85 | 1.28 | 9.68E-01 |
| Female | Female_Insomnia_Daytime_Dozing_1.00 | -0.03 | 0.97 | 0.79 | 1.18 | 9.58E-01 |
| Male | Male_Insomnia_Sleep_Duration_1.00 | -0.02 | 0.98 | 0.85 | 1.12 | 9.23E-01 |
| Female | Female_PTSD_1.00 | -0.04 | 0.97 | 0.77 | 1.20 | 9.15E-01 |
| Female | Female_Worry_Subcluster_1.00 | -0.03 | 0.98 | 0.80 | 1.19 | 9.42E-01 |
| Male | Male_Insomnia_Morningness_1.00 | -0.02 | 0.98 | 0.86 | 1.13 | 9.37E-01 |
| Male | Male_Insomnia_1.00 | 0.02 | 1.02 | 0.88 | 1.17 | 9.10E-01 |
| Male | Male_Suicide_Death_1.00 | 0.01 | 1.02 | 0.88 | 1.17 | 8.87E-01 |
| Female | Female_Insomnia_Morningness_1.00 | -0.01 | 0.99 | 0.81 | 1.21 | 9.88E-01 |
| Female | Female_Insomnia_Ease_of_Getting_Up_1.00 | 0.00 | 1.00 | 0.82 | 1.22 | 9.73E-01 |

# References

1 Nurnberger, J. I., Jr. *et al.* Diagnostic interview for genetic studies. Rationale, unique features, and training. NIMH Genetics Initiative. *Archives of general psychiatry* **51**, 849-859; discussion 863-844 (1994).

2 Das, S. *et al.* Next-generation genotype imputation service and methods. *Nature genetics* **48**, 1284-1287, doi:10.1038/ng.3656 (2016).

3 Martin, J. *et al.* A Genetic Investigation of Sex Bias in the Prevalence of Attention-Deficit/Hyperactivity Disorder. *Biological psychiatry* **83**, 1044-1053, doi:10.1016/j.biopsych.2017.11.026 (2018).

4 Demontis, D. *et al.* Discovery of the first genome-wide significant risk loci for attention deficit/hyperactivity disorder. *Nature genetics* **51**, 63-75, doi:10.1038/s41588-018-0269-7 (2019).

5 Otowa, T. *et al.* Meta-analysis of genome-wide association studies of anxiety disorders. *Molecular psychiatry* **21**, 1391-1399, doi:10.1038/mp.2015.197 (2016).

6 Jansen, P. R. *et al.* Genome-wide analysis of insomnia in 1,331,010 individuals identifies new risk loci and functional pathways. *Nature genetics* **51**, 394-403, doi:10.1038/s41588-018-0333-3 (2019).

7 Nievergelt, C. M. *et al.* International meta-analysis of PTSD genome-wide association studies identifies sex- and ancestry-specific genetic risk loci. *Nature communications* **10**, 4558, doi:10.1038/s41467-019-12576-w (2019).

8 Mullins, N. *et al.* GWAS of Suicide Attempt in Psychiatric Disorders and Association With Major Depression Polygenic Risk Scores. *The American journal of psychiatry* **176**, 651-660, doi:10.1176/appi.ajp.2019.18080957 (2019).

9 Nagel, M. *et al.* Meta-analysis of genome-wide association studies for neuroticism in 449,484 individuals identifies novel genetic loci and pathways. *Nature genetics* **50**, 920-927, doi:10.1038/s41588-018-0151-7 (2018).

10 Euesden, J., Lewis, C. M. & O'Reilly, P. F. PRSice: Polygenic Risk Score software. *Bioinformatics* **31**, 1466-1468, doi:10.1093/bioinformatics/btu848 (2015).

11 Docherty, A. R. *et al.* Genome-Wide Association Study of Suicide Death and Polygenic Prediction of Clinical Antecedents. *The American journal of psychiatry* **177**, 917-927, doi:10.1176/appi.ajp.2020.19101025 (2020).

12 R: A Language and Environment for Statisitcal Computing (R Foundation for Statistical Computing, Vienna, Austria, 2020).

13 Forestplot: Advanced Forest Plot Using 'grid' Graphics (2020).

14 Wickham, H. in *Use R!,* 1 online resource (XVI, 260 pages 232 illustrations, 140 illustrations in color (Springer International Publishing : Imprint: Springer,, Cham, 2016).

15 Sanders, A. R. *et al.* The Internet-based MGS2 control sample: self report of mental illness. *The American journal of psychiatry* **167**, 854-865, doi:10.1176/appi.ajp.2010.09071050 (2010).
